# Enforced tethering elongates the cortical endoplasmic reticulum and limits store-operated Ca^2+^ entry

**DOI:** 10.1242/jcs.259313

**Published:** 2022-03-30

**Authors:** Christopher Henry, Amado Carreras-Sureda, Nicolas Demaurex

**Affiliations:** Department of Cell Physiology and Metabolism, University of Geneva, Geneva 1211, Switzerland

**Keywords:** Ca^2+^ signaling, Electron microscopy, Innate immunity, Ion channels, Membrane contact sites, Muscle physiology

## Abstract

Recruitment of STIM proteins to cortical endoplasmic reticulum (cER) domains forming membrane contact sites (MCSs) mediate the store-operated Ca^2+^ entry (SOCE) pathway essential for human immunity. The cER is dynamically regulated by STIM and tethering proteins during SOCE, but the ultrastructural rearrangement and functional consequences of cER remodeling are unknown. Here, we express natural (E-Syt1 and E-Syt2) and artificial (MAPPER-S and MAPPER-L) protein tethers in HEK-293T cells and correlate the changes in cER length and gap distance, as measured by electron microscopy, with ionic fluxes. We found that native cER cisternae extended during store depletion and remained elongated at a constant ER-plasma membrane (PM) gap distance during subsequent Ca^2+^ elevations. Tethering proteins enhanced store-dependent cER expansion, anchoring the enlarged cER at tether-specific gap distances of 12-15 nm (E-Syts) and 5-9 nm (MAPPERs)*.* Cells with artificially extended cER had reduced SOCE and reduced agonist-induced Ca^2+^ release. SOCE remained modulated by calmodulin and exhibited enhanced Ca^2+^-dependent inhibition. We propose that cER expansion mediated by ER-PM tethering at a close distance negatively regulates SOCE by confining STIM-ORAI complexes to the periphery of enlarged cER sheets, a process that might participate in the termination of store-operated Ca^2+^ entry.

## Abstract (precis)

**Summary:** ER-PM tethering at close distance limits Ca^2+^ entry by confining STIM-ORAI complexes to the periphery of contact sites.

## INTRODUCTION

Store-operated Ca^2+^ entry (SOCE) is a ubiquitous signaling mechanism that plays an essential role in immunity and muscle function in humans by mediating long-lasting Ca^2+^ signals that control gene expression and cell proliferation (Kiviluoto et al., 2011; Vaeth et al., 2017). Upon agonist stimulation, G-protein-coupled receptors generate inositol trisphosphate (InsP3) that mobilize Ca^2+^ from the endoplasmic reticulum (ER). The ensuing Ca^2+^ depletion of the ER is sensed by the ER membrane-resident protein stromal interaction molecule 1 (STIM1) (Liou et al., 2005; Roos et al., 2005; Zhang et al., 2005) via its luminal EF-hand and sterile α-motif (SAM) domains (Stathopulos et al., 2006). This initiates a conformational change that releases an intramolecular clamp between cytosolic coiled-coil domains, leading to STIM1 extension and oligomerization (Hirve et al., 2018; Schober et al., 2019). The switch into an elongated conformation unveils the STIM1 channel-activating domain (CAD) and exposes a lysine-rich polybasic C-terminal tail that binds to negatively charged lipids (Stathopulos and Ikura, 2010; Stathopulos et al., 2006; Zhang et al., 2005). Exposure of the CAD and tail of STIM1 promote the translocation of STIM1 oligomers to cortical ER (cER) regions and enable STIM1 to trap and gate the ORAI family of Ca^2+^-selective channels (Feske et al., 2006; Vig et al., 2006; Zhang et al., 2006). The flow of Ca^2+^ ions across ORAI channels sustains long-lasting cytosolic Ca^2+^ signals and enables the refilling of ER Ca^2+^ stores by sarco/ER Ca^2+^-ATPase (SERCA) pumps (Chang et al., 2013; Malli et al., 2007; Mullins et al., 2009), thereby terminating SOCE. Excessive Ca^2+^ entry is prevented by fast (in the order of milliseconds) and slow (in the order of seconds) Ca^2+^-dependent inactivation (CDI) mechanisms mediated in part by calmodulin and SARAF, which interact with STIM-ORAI complexes to regulate channel activity (Bhardwaj et al., 2020; Jardin et al., 2018; Palty et al., 2012).

The SOCE process relies on the generation and maintenance of membrane contact sites (MCSs) between the ER and plasma membrane (PM) (Gudlur et al., 2020) that enable direct interaction between STIM and ORAI proteins by maintaining an appropriate gap distance ranging from 10 to 30 nm between the cER and the PM (Scorrano et al., 2019; Wu et al., 2017). The morphology of the MCSs evolves during the SOCE process as STIM and other tethering proteins are recruited to the cER and reversibly bind to PM lipids and interact with cognate PM proteins. In HeLa cells, the surface covered by MCSs increases ∼5-fold (from 0.23% to 1.24%) upon Ca^2+^ depletion, a remodeling that is recapitulated by STIM1 expression in cells with replete stores (Orci et al., 2009). Expression of STIM1, but not of the muscle-specific STIM1L isoform, increases MCS coverage in mouse embryonic fibroblasts (MEF) and in myoblasts, indicating that distinct STIM1 isoforms have different membrane-shaping functions (Sauc et al., 2015). Other tethering proteins were also reported to participate in the dynamic formation of MCSs during SOCE. Junctate (encoded by *ASPH*) stabilizes the intracellular MCSs generated by STIM1, thereby contributing to prophagocytic Ca^2+^ signals by recruiting inositol-trisphosphate receptors (InsP3R) to phagosomes (Guido et al., 2015). The extended synaptotagmin-1 (E-Syt1) is recruited to the cER upon Ca^2+^ elevations (Giordano et al., 2013; Idevall-Hagren et al., 2015), and its expression induces a shortening of the ER-PM gap distance from 21.8 nm to 14.8 nm in COS-7 cells (Fernandez-Busnadiego et al., 2015). The two other E-Syt isoforms, E-Syt2 and E-Syt3 are already localized within MCSs at rest and do not promote gap shortening upon Ca^2+^ entry (Giordano et al., 2013). The maintenance of an appropriate ER-PM gap distance at MCSs has been proposed to regulate SOCE by enabling the access of additional molecular components to STIM1-ORAI1 complexes (Varnai et al., 2007), whereas the Ca^2+^-dependent E-Syt1 recruitment has been proposed to facilitate the recruitment of the phosphatidylinositol transfer protein Nir2 (also known as PITPNM1) to replenish PM phosphatidylinositol 4,5-bisphosphate (PIP2) following receptor-induced hydrolysis (Chang et al., 2013). A subsequent super-resolution microscopy study showed that the Ca^2+^-dependent recruitment of E-Syt1 stabilizes ring-shaped MCSs forming around STIM1-ORAI1 MCSs, a process proposed to facilitate local ER Ca^2+^ replenishment (Kang et al., 2019).

The importance of MCSs in Ca^2+^ signaling and lipid trafficking motivated several groups to develop artificial ER-PM tether proteins to label and manipulate MCSs. Using the transmembrane domain of STIM for ER retention and the polybasic motif of the small G protein Rit for constitutive binding to PM phosphoinositides, the group of Jen Liou developed a genetically encoded marker for ER-PM junctions termed ‘MAPPER’ for ‘membrane-attached peripheral ER’ (Chang et al., 2013). Two versions of MAPPER were generated: a long version (MAPPER-L) bearing flexible cytosolic linkers meant to preserve the endogenous 10-25 nm ER-PM gap distance, and a short version (MAPPER-S) designed to restrict the gap distance of the labeled junctions to 10 nm. MAPPER-L was found to be enriched in the cER independently of the ER Ca^2+^ concentration and STIM1 and E-Syt1 translocated into MAPPER-L-labeled structures following agonist stimulation or Ca^2+^ store depletion. MAPPER-L labeling did not perturb the endogenous ER-PM junctions and did not impact SOCE whereas E-Syt1 recruitment to MCSs shortened the ER-PM gap without altering the sizes and shapes of individual junctions (Chang et al., 2013). MAPPERs were subsequently used to study the distribution of ER proteins populating ER-PM junctions (Thillaiappan et al., 2017; Zewe et al., 2018) and to study MCS dynamics (Poteser et al., 2016). It was revealed that the junctional ER expands during SOCE in RBL-2H3 cells in a process that requires external Ca^2+^ (Poteser et al., 2016). In several instances however, expression of mCherry-tagged MAPPER-L induced the formation of abnormally large MCSs (Besprozvannaya et al., 2018; Thillaiappan et al., 2017), suggesting that MAPPER-L is not simply labeling cER structures but also modifying their morphology.

To better understand the role of MCS morphology during SOCE, we expressed the natural and artificial tether proteins E-Syt1, E-Syt2, MAPPER-S, and MAPPER-L in HEK-293 T cells and measured the ER-PM gap distance and cER length by electron microscopy (EM). Quantification of the cER morphometric parameters revealed that very large cER structures were generated by the expression of E-Syt2 or MAPPER-L during ER Ca^2+^ depletion, a process that was partly reverted upon Ca^2+^ entry. Recordings of the Ca^2+^ indicator Fura-2 revealed that SOCE was reduced in cells expressing either E-Syt2 or MAPPER-L, suggesting that the cER length but not the ER-PM gap distance negatively regulates SOCE efficiency.

## RESULTS

### Depletion of Ca^2+^ stores enlarges native cortical ER sheets

**Fig. 1.**
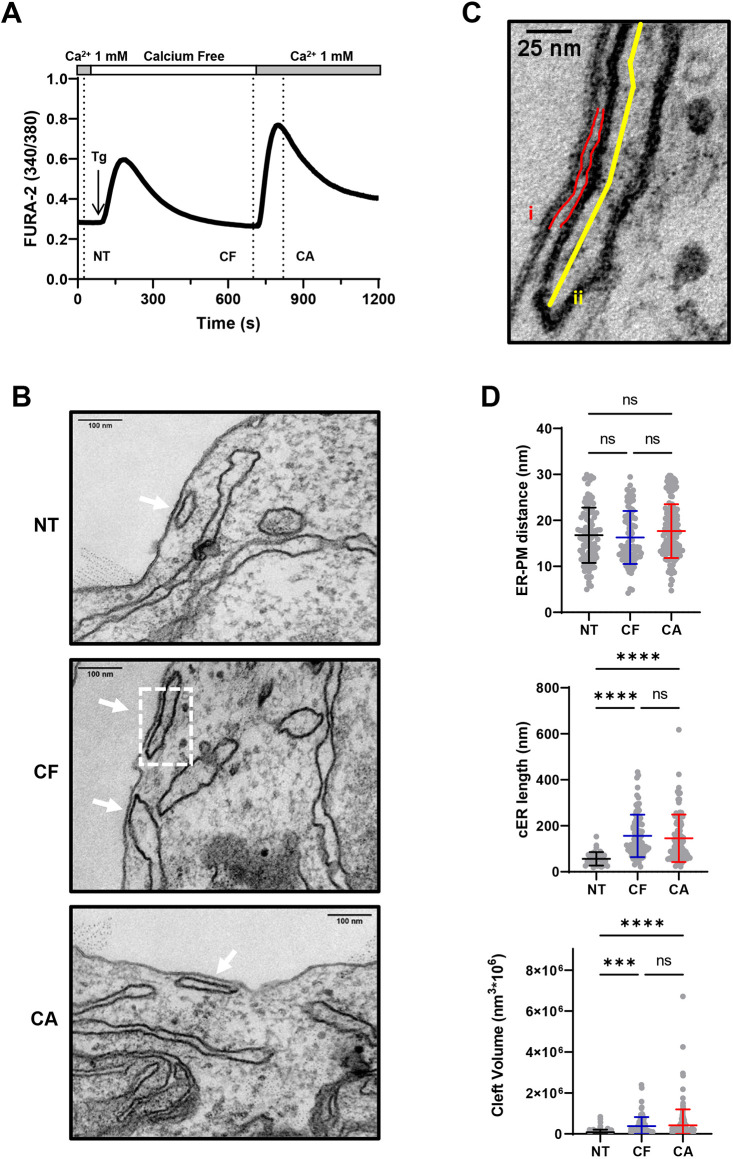
**Ca^2+^ depletion promotes the elongation of the native cortical ER.** (A) Ca^2+^ recording illustrating the conditions used for EM analysis during the Tg-readmission protocol. HEK-293 T cells were either untreated (NT), treated for 10 min with 1 μM Tg in Ca^2+^-free (CF) medium to deplete ER Ca^2+^ stores or subsequently re-exposed to 1 mM Ca^2+^ for 2 min to allow Ca^2+^ entry (CA). They were then fixed, embedded in Epon resin, sectioned, and observed for Fura-2 fluorescence. (B) Representative electron micrographs showing the morphology of cortical ER sheets at membrane contact sites (white arrows) in the three conditions (*n*=3 slices from one sample). Scale bars: 100 nm. (C) Zoomed image of a cER sheet seen in the dotted box from the CF condition in B. The lines drawn to extract the ER-PM distance (i, red) and cER length (ii, yellow) are shown. Scale bar: 25 nm. (D) Quantification of the ER-PM gap distance (top), cER length (middle) and cleft volume (bottom) in the three conditions. Cleft volume was calculated from the cER gap and length values assuming a cylindric shape. Data are mean±s.d. of 105, 89 and 146 quantified structures; ns, not significant; ****P<0.0001; ***P<0.001 (one-way ANOVA with Tukey's post-hoc test for the three parameters).

To resolve the ultrastructure of the MCSs forming during Ca^2+^ store depletion, we analyzed by EM the morphology of individual cER structures in cells fixed and embedded in Epon resin *in situ*. This procedure optimally preserves cellular architecture and maintains the orientation of cells relative to the substrate. Cells were divided into three groups: (1) non-treated cells (NT), (2) cells exposed to 1 µM thapsigargin (Tg) for 10 min in the absence of Ca^2+^ to deplete ER Ca^2+^ stores (Ca^2+^ free, CF) and favor the formation of STIM1-mediated ER-PM MCSs, and (3) cells treated with Tg as in (2) and re-exposed to 1 mM Ca^2+^ for 2 min to enable Ca^2+^ influx (Ca^2+^-containing medium, CA) and evaluate the impact of Ca^2+^ elevations on MCS morphology (Fig. 1A). cER structures were identified on the EM images as ER sheets located <30 nm from a continuous segment of the PM (Fig. 1B), and each contiguous cER unit was manually outlined to determine two parameters: the ER-PM gap distance and the length of single cER units. The ER-PM distance was obtained from the average pixel-to-pixel distance between the PM and the apposed ER membrane and the cER length by outlining the cER largest diameter (Fig. 1C). We found that in non-treated HEK-293 T cells the average gap distance and cER length were 17 nm and 66 nm, respectively (Fig. 1D; Table S1). In cells treated with Tg, a massive elongation of the cER was observed, the average length increasing by 2.3-fold, from 66 to 156 nm, while the ER-PM gap distance remained unchanged (Fig. 1D; Tables S1 and S2). We then calculated the volume of the cytosolic cleft contained between the cER and PM membranes, assuming that cER sheets form cylindric structures close to the PM (Fig. S1). The cleft volume increased by 4.8-fold upon Tg treatment (Fig. 1D; Tables S1 and S2). Interestingly, the gap distance, cER length, and cleft volume were not impacted by the readmission of Ca^2+^ to cells treated with Tg (Fig. 1D). These ultrastructural observations indicate that cER are dynamic structures that elongate during store depletion, as previously shown for cells overexpressing STIM proteins (Orci et al., 2009; Sauc et al., 2015; Wu et al., 2006), but that the ER-PM gap distance remains constant during SOCE activation and Ca^2+^ entry. Furthermore, the length of native cER structures and their gap distance are not affected by cytosolic Ca^2+^ elevations occurring during SOCE.

### Expression of E-Syts and MAPPERs enhances cER expansion upon Ca^2+^ store depletion

To establish how ER-PM tethering impacts the cER structure and dynamics we transiently transfected HEK-293 T cells with a plasmid expressing GFP-tagged versions of natural (E-Syt1 or E-Syt2) or artificial (MAPPER-S or MAPPER-L) tether proteins. We first verified by total internal reflection fluorescence (TIRF) microscopy that these tether proteins are recruited to regions of the ER close to the PM in cells exposed to Tg. GFP–E-Syts and GFP–MAPPERs were detected in the TIRF plane as punctate fluorescent structures of 0.3 µm^2^ that covered 5-10% of the PM surface without significant differences between tether proteins (Fig. S2). These data indicate that all four tether proteins accumulate in the TIRF plane into structures of similar apparent size when observed by optical microscopy.

**Fig. 2.**
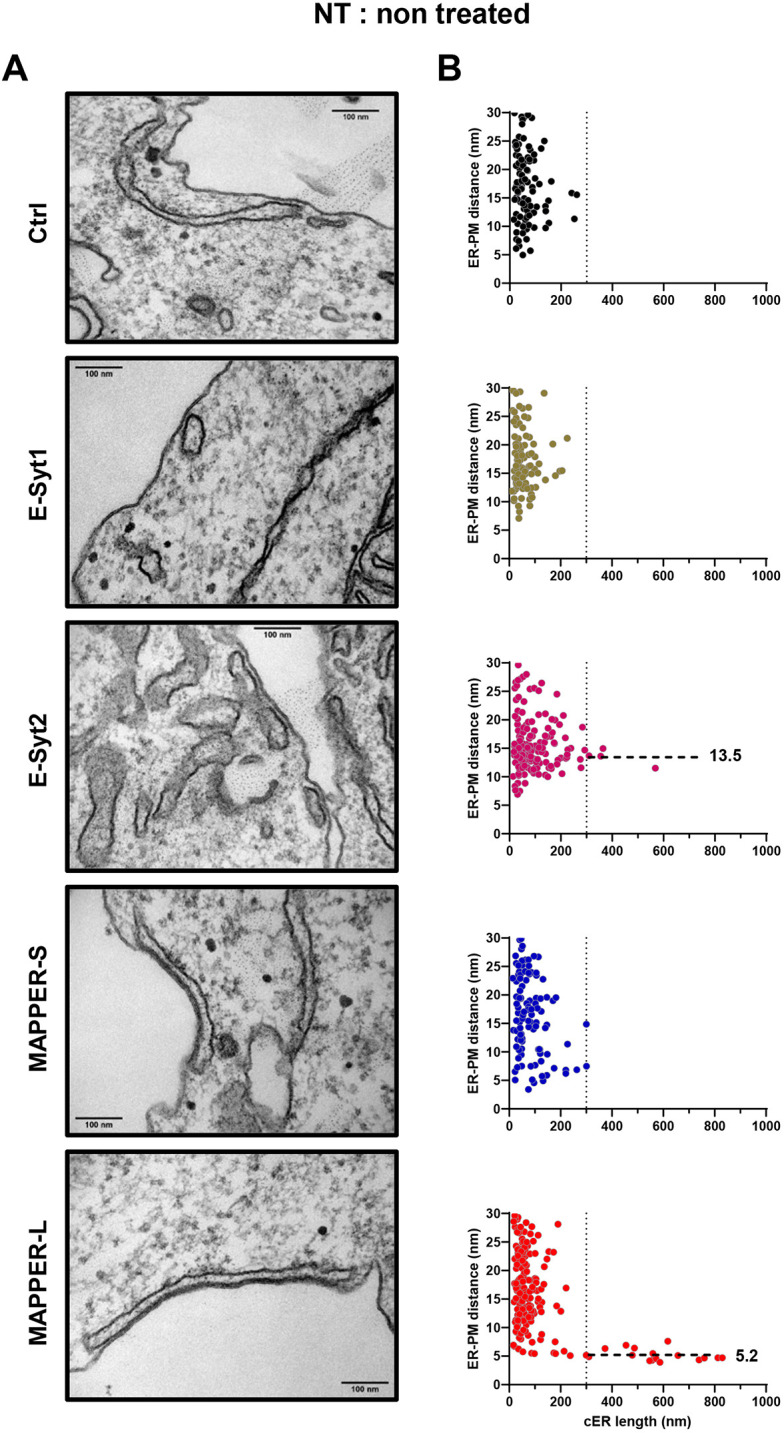
**Effect of expression of E-Syts and MAPPERs on cER morphology in non-treated HEK-293 T cells.** (A) Representative electron micrographs of cER sheets in cells expressing the indicated ER-PM tethers, before store depletion (*n*=3 slices from one sample). Scale bars: 100 nm. (B) Scatter plots of ER-PM gap distances as a function of cER length in each condition. E-Syt2 and MAPPER-L expression induced the formation of elongated cER sheets anchored at a fixed distance from the PM, dashed lines and values (in nm) indicate the average gap distance at cER length >300 nm (indicated by vertical dotted lines). The corresponding quantitative analysis is presented in Tables S1 and S2.

**Fig. 3.**
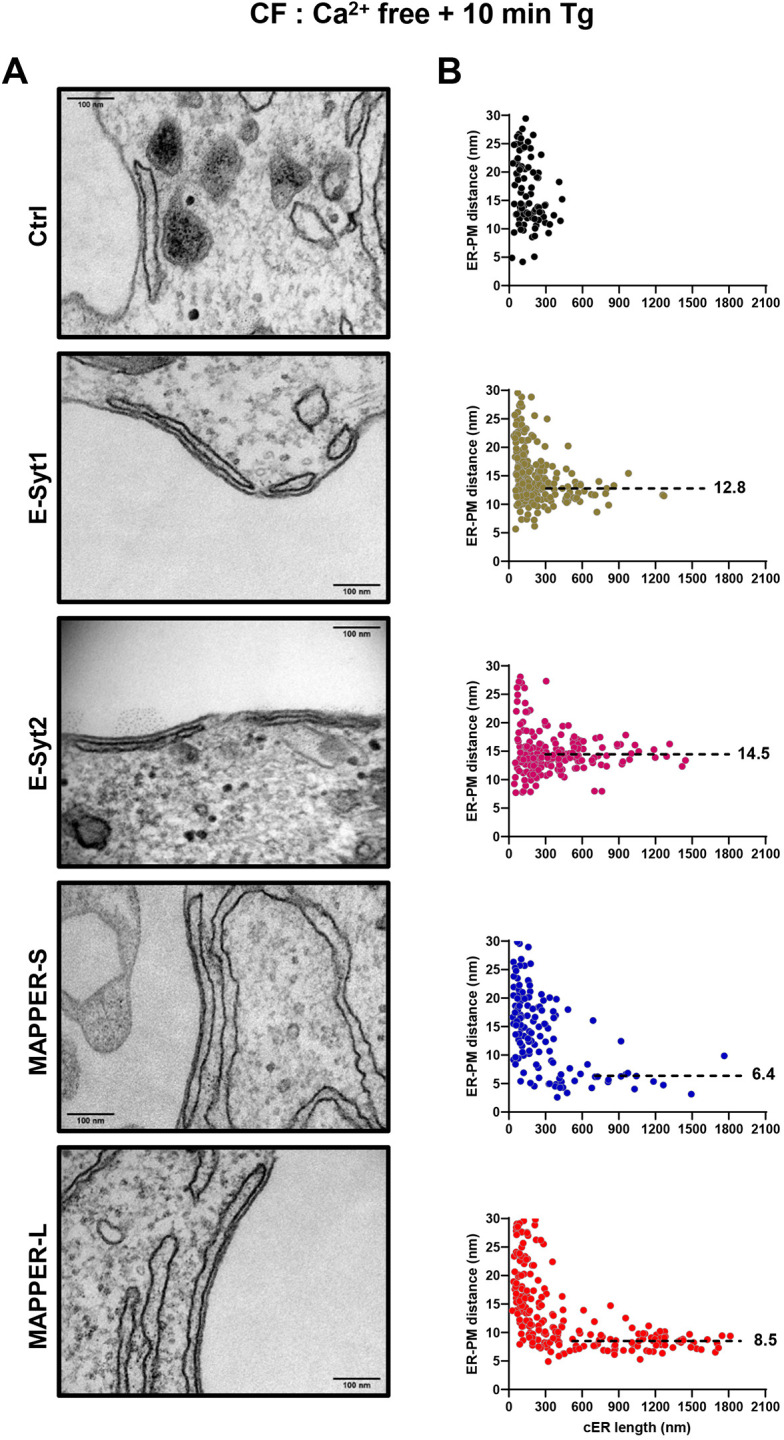
**Expression of E-Syts and MAPPERs augments the cER elongation during Ca^2+^ store depletion.** (A) Representative electron micrographs of cER sheets in cells expressing the indicated ER-PM tethers, 10 min after Tg addition in Ca^2+^-free medium (*n*=3 slices from one sample). Scale bars: 100 nm. (B) Scatter plots of ER-PM gap distances as a function of cER length in each condition. Expression of each tether induced the formation of elongated cER sheets anchored at a fixed distance from the PM (dashed lines on the asymptotic part of the distribution starting at 300 nm for E-Syts, at 700 nm for MAPPER-S and at 600 nm for MAPPER-L; mean values in nm). The corresponding quantitative analysis is presented in Tables S1 and S2.

**Fig. 4.**
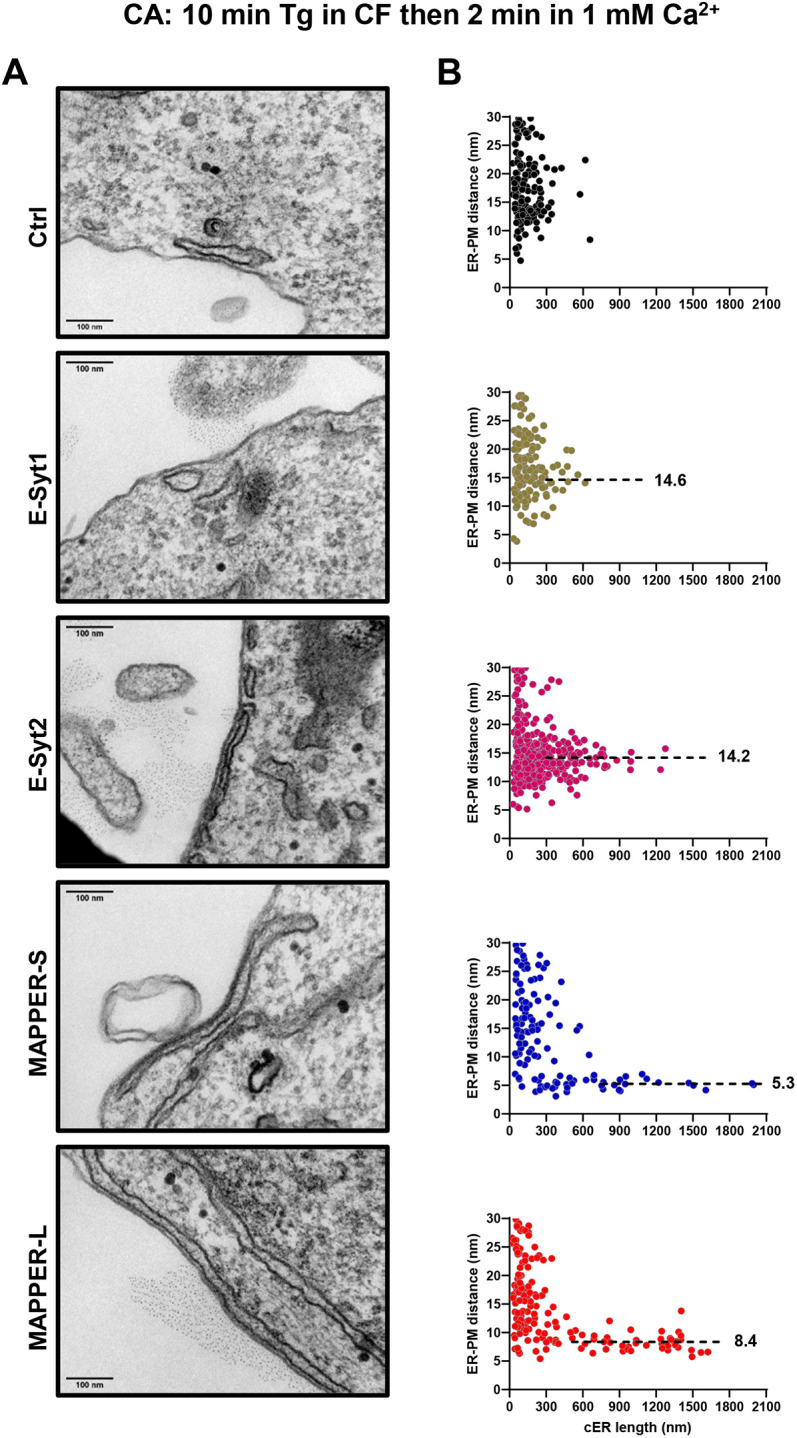
**The cER elongation persists in E-Syts and MAPPERs cells after store-operated Ca^2+^ influx.** (A) Representative electron micrographs of cER sheets in cells expressing the indicated ER-PM tethers, 2 min after Ca^2+^ readmission (*n*=3 slices from one sample). Scale bars: 100 nm. (B) Scatter plots of ER-PM gap distances as a function of cER. Elongated cER sheets persisted in cells expressing the tethers, anchored at a fixed distance from the PM (dotted lines; mean values in nm). The corresponding quantitative analysis is presented in Tables S1 and S2.

We then assessed the impact of the expression of E-Syts or MAPPERs on MCS ultrastructure. Transfected cells were sorted by flow cytometry to ensure that equal amounts of tether proteins were expressed (Fig. S3). Cells with low to moderate overexpression were selected to avoid the formation of artificial cortical ER structures in the absence of store depletion. In resting cells, the average gap distance and cER length were not impacted by the expression of any of the tether proteins (Tables S1 and S2), but MAPPER-L and E-Syt2 induced the formation of cER cisternae longer than 300 nm (Fig. 2A). These elongated cER structures stabilized at specific gap distances of 5.2 nm and 13.5 nm, respectively, clearly apparent on the gap versus length scatter plots (Fig. 2B). Following Ca^2+^ store depletion with Tg, the elongation of the cER was greatly potentiated in cells expressing any of the tether proteins, the average length increasing by 4.5-fold in cells expressing MAPPER-L, from 117 to 521 nm (Fig. 3; Table S1). Interestingly, as the length of individual cER structures increased, the gap distance decreased to values specific for each family of tether protein, of 12-15 nm for the E-Syts and 5-9 nm for the MAPPERs (Fig. 3B). Ca^2+^ store depletion was associated with a shortening of the averaged ER-PM distance in cells expressing all tethers except E-Syt2, the averaged gap decreasing from 17.3 nm to 14.9 nm in cells expressing E-Syt1 upon Tg addition (Tables S1 and S2). After readmission of Ca^2+^ to enable Ca^2+^ influx, the cER length decreased significantly in cells expressing all tethers except MAPPER-S, while the gap distance returned to pre-stimulatory values in cells expressing E-Syt1 (Fig. S4, Tables S1 and S2). In cells expressing the other tethers, the gap distance remained unchanged and cER cisternae longer than 300 nm were still clustered at gap distances specific for each tether (Fig. 4). As the cER expanded, the cleft volume increased upon Tg treatment in all conditions by up to 16.9-fold, with the largest volumes observed in cells expressing E-Syt2 and MAPPER-L (Fig. S4, Tables S1 and S2). These observations indicate that ER-PM tethering enhances cER expansion during store depletion by anchoring elongated cER cisternae at fixed gap distances, and that this elongation process is partly reversed during the Ca^2+^ entry phase. The expanded cER sheets are anchored at 12-15 nm from the PM by E-Syts and at 5-9 nm by MAPPERs and these gap distances are minimally affected by changes in cytosolic Ca^2+^ concentration.

### Effect of ER-PM tethering on Ca^2+^ store homeostasis and SOCE

**Fig. 5.**
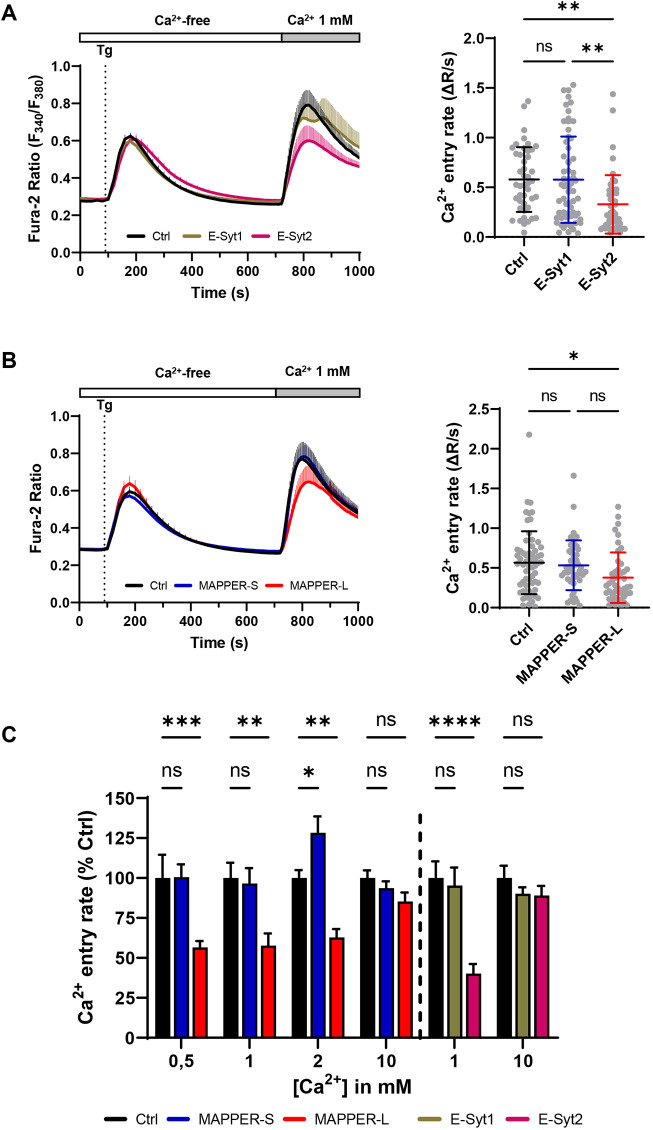
**E-Syt2 and MAPPER-L expression inhibits store-operated Ca^2+^ entry.** (A) Left, averaged Fura-2 recordings of Ca^2+^ elevations evoked by the Tg-readmission protocol in cells expressing the E-Syt1 (*n*=63), E-Syt2 (*n*=49) or a control plasmid (*n*=44). Right, quantification of the Ca^2+^ entry rates. (B) Averaged Ca^2+^ recordings (left) and Ca^2+^ entry rates (right) in cells expressing the MAPPER-S (*n*=48), MAPPER-L (*n*=45) or a control plasmid (*n*=62). (C) Ca^2+^ entry rates recorded at varying Ca^2+^ concentration in cells expressing the indicated tether (left to right *n*=50, 92, 97, 62, 48, 45, 80, 70, 48, 70, 110, 72, 44, 63, 49, 39, 91 and 75 cells). Data are mean±s.d. (A,B) or s.e.m. (C) of cells from 5-9 independent recordings; ns: not significant; *P<0.05; **P<0.01; ***P<0.001; ****P<0.0001 [one-way ANOVA with Tukey's (A,B) or Dunnett's (C) post-hoc test].

To assess whether the cER expansion facilitated by ER tethering impacts SOCE, we measured the cytosolic Ca^2+^ elevations during the Tg-readmission protocol with Fura-2 in cells expressing E-Syts or MAPPERs at equivalent levels. The Ca^2+^ elevations evoked by Tg in Ca^2+^-free medium were comparable in all conditions, indicating that the global ER Ca^2+^ content is not impacted by ER tethering (Fig. S5A). The SOCE responses, however, were significantly reduced by ER tethering and the Ca^2+^ entry rates measured following readmission of 1 mM Ca^2+^ were decreased by 60% and 40% in cells expressing E-Syt2 and MAPPER-L, respectively (Fig. 5A,B). We confirmed that SOCE was still reduced in cells expressing E-Syt2 or MAPPER-L after they were FACS sorted for the EM experiment (Fig. S5B). Since these two protein tethers induce a maximal cER expansion that is Ca^2+^ dependent, we tested whether the SOCE defect was also Ca^2+^ dependent by varying the concentration of Ca^2+^ re-added following Tg exposure. The SOCE defect associated with MAPPER-L expression persisted when the Ca^2+^ concentration was decreased to 0.5 mM or increased to 2 mM Ca^2+^, and lost when Ca^2+^ concentration was increased to 10 mM (Fig. 5C). Interestingly, at 2 mM Ca^2+^ we observed a slight increase of the Ca^2+^ entry rate when MAPPER-S was expressed. These data indicate that the expression of tethering proteins that promote cER expansion inhibits SOCE, and that the associated ultrastructural and functional effects are both Ca^2+^ dependent.

**Fig. 6.**
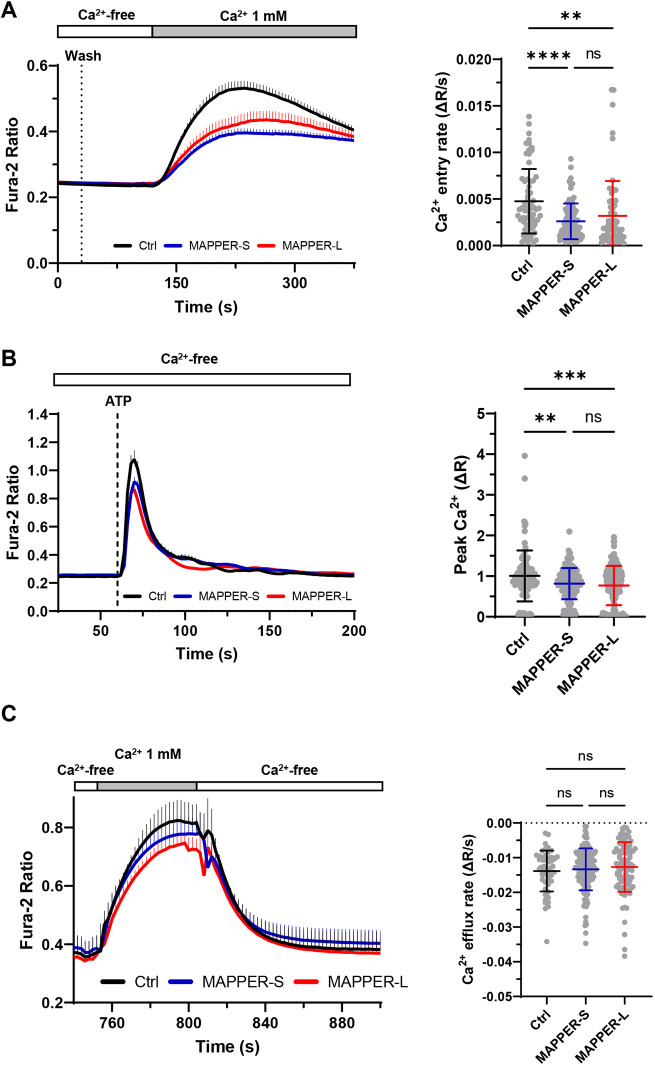
**Expression of MAPPERs perturbs ER Ca^2+^ store homeostasis.** (A) Averaged Ca^2+^ recordings (left) and Ca^2+^ entry rates (right) in cells expressing MAPPER-S (*n*=86), MAPPER-L (*n*=64) or a control plasmid (*n*=68), transiently exposed to the reversible SERCA inhibitor CPA to enable store refilling during Ca^2+^ readmission. Wash: removal of CPA. Expression of both MAPPER tethers decreased Ca^2+^ entry rates in these conditions. (B) Averaged Ca^2+^ recordings of cells expressing MAPPER-S (*n*=166), MAPPER-L (*n*=153) or a control plasmid (*n*=98), exposed to 100 µM ATP in Ca^2+^-free medium. Right, quantification of the peak amplitude of the evoked responses. (C) Averaged Ca^2+^ recordings (left) and Ca^2+^ extrusion rates (right) of cells expressing MAPPER-S (*n*=134), MAPPER-L (*n*=98) or a control plasmid (*n*=52). Ca^2+^ was removed 2 min after readmission to assess PMCA-mediated Ca^2+^ extrusion. Data are mean±s.d. of cells from 6-9 independent recordings; ns: not significant; **P<0.01; ***P<0.001; ****P<0.0001 (one-way ANOVA with Tukey's post-hoc test).

To clarify the Ca^2+^ signaling defect associated with ER tethering, we expressed the two MAPPER constructs in different cell types. The SOCE defect associated with MAPPER-L expression was recapitulated in MEF cells, whereas a reduced SOCE was observed in HeLa cells expressing either the long or short MAPPER constructs (Fig. S6). This indicates that the SOCE defect associated with ER tethering is not restricted to a specific cell type or to proteins tethering the ER at a specific gap distance, but is likely related to the cER expansion. To better assess the contribution of local Ca^2+^ fluxes in the functional effects of the MAPPERs, we then used the reversible SERCA inhibitor cyclopiazonic acid (CPA) to allow the refilling of depleted ER Ca^2+^ stores during Ca^2+^ readmission. SOCE was equally reduced by the two MAPPERs when CPA was used to transiently deplete ER Ca^2+^ stores in HEK-293 T cells (Fig. 6A), uncovering a defect associated with MAPPERs that was not observed when Tg was used to irreversibly deplete Ca^2+^ stores (Fig. 5B). To test whether the reduced SOCE impacts physiological Ca^2+^ fluxes, we then exposed cells to the InsP3-generating agonist ATP to promote the rapid release of Ca^2+^ from ER stores. The amplitude of the ATP-induced Ca^2+^ elevations recorded in the absence of Ca^2+^ was reduced by ∼20% when either of the two MAPPER constructs was expressed (Fig. 6B). These data indicate that the expression of tethering proteins that promote cER expansion reduces the ability of cells to release Ca^2+^ from InsP3-sensitive stores.

**Fig. 7.**
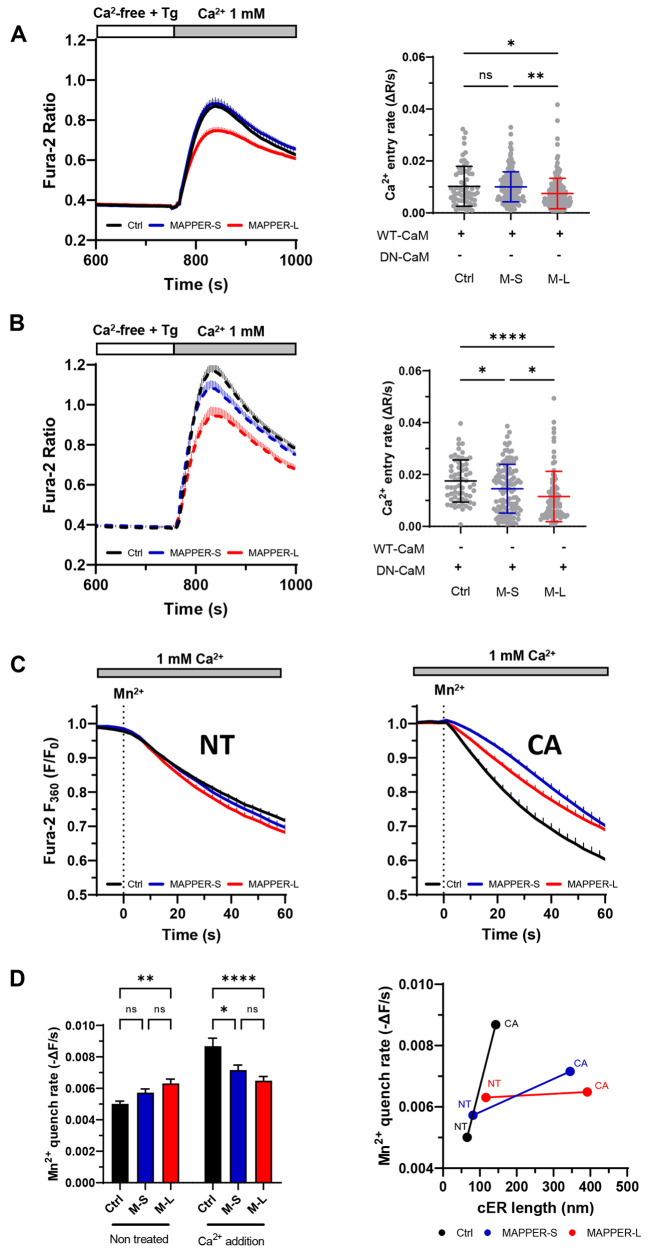
**Expression of MAPPERs inhibits SOCE independently of calmodulin.** (A,B) Averaged Ca^2+^ recordings (left) and Ca^2+^ entry rates (right) in cells expressing MAPPER-S (M-S) (*n*=174), MAPPER-L (M-L) (*n*=183) or a control plasmid (*n*=66) together with wild-type WT-CaM (A) or dominant-negative DN-CaM (B). DN-CaM positively modulated SOCE independently of MAPPERs expression. Data are mean±s.e.m. of (M-S=183; M-L=85; Ctrl=109) cells from 3-11 independent recordings (C) Averaged Mn^2+^ quench recordings in cells expressing MAPPERs or a control plasmid before stimulation (left, NT), following Ca^2+^ readmission after store depletion (right, CA). (D) Statistical evaluation of the Mn^2+^ quenching rates (left panel) (left to right *n*=406, 298, 269, 171, 187, 181). Mn^2+^ quench rates as a function of cER length for the NT and CA conditions (right). MAPPERs expression caused massive cER enlargement without enhancing SOCE. Data are mean±s.d. (A,B) or s.e.m. (D) of cells from 5-12 independent recordings; ns: not significant; *P<0.05; **P<0.01; ****P<0.0001 (one-way ANOVA with Tukey's post-hoc test for A, B, D).

To get an insight into the underlying mechanism, we then investigated the impact of ER tethering on cytosolic Ca^2+^ extrusion by removing Ca^2+^ shortly after its readmission to cells treated with Tg, a procedure that isolates the activity of plasma membrane extrusion mechanisms (Frieden et al., 2005). The Ca^2+^ concentration decreased rapidly following Ca^2+^ removal and the decay rates were not altered by the expression of the MAPPER proteins (Fig. 6C). This indicates that the cER expansion associated with ER tethering does not impact Ca^2+^ efflux at the PM, a process mediated by plasma membrane Ca^2+^ ATPase (PMCA) (Brini and Carafoli, 2009). The increased ER-PM cleft volume associated with ER tethering could promote the accumulation of STIM1-ORAI1 interactors that negatively regulate SOCE. To test this possibility, we overexpressed wild-type (WT) or dominant-negative (DN; a Ca^2+^-insensitive calmodulin mutant bearing aspartate to alanine mutations in all four EF-hands) calmodulin (CaM) constructs together with the MAPPER tethers. Expression of DN-CaM increased SOCE (Fig. 7A,B), confirming that CaM negatively regulates STIM1-ORAI1 interactions as previously reported (Singh et al., 2012; Bhardwaj et al., 2020). Regardless of WT- or DN-CaM expression, SOCE remained reduced in cells expressing MAPPER-L (Fig. 7B), indicating that the negative regulation of SOCE associated with ER tethering is not relieved by preventing the activity of calmodulin.

To verify that cER tethering altered the permeability of SOCE channels, we added the divalent cation Mn^2+^, which permeates SOCE channels and quenches the fluorescence of Fura-2, measured at the isosbestic wavelength of 360 nm. The basal rates of Mn^2+^ quenching were slightly increased by MAPPER-L expression when Mn^2+^ was added in Ca^2+^-containing medium (Fig. 7C,D), but were comparable when Mn^2+^ was added in nominal Ca^2+^-free medium without EGTA to avoid Mn^2+^ chelation (Fig. S7A). In non-transfected cells, Tg increased Mn^2+^ quench rates by 2.4-fold in nominal Ca^2+^-free medium, consistent with SOCE activation (Fig. S7A). Following readmission of 1 mM Ca^2+^ the quench rates remained elevated in control cells but were close to basal levels in cells expressing the MAPPERs (Fig. 7C,D). Correlation of Mn^2+^ quench rates and cER lengths measured before Tg addition and following Ca^2+^ readmission confirmed that Mn^2+^ entry increased proportionally to cER length in non-transfected cells but not in cells expressing MAPPER-L despite a massive cER expansion (Fig. 7D). These data indicate that the cER elongation potentiated by ER-PM tethering does not enhance store-operated cation entry. To identify the route of Mn^2+^ entry, we measured Mn^2+^ quenching in HEK-293 T cells lacking all three ORAI isoforms [triple knockout (TKO) cells]. Fura-2 quenching by Mn^2+^ was abrogated in TKO cells and restored by ORAI1 re-expression (Fig. S7B), indicating that Mn^2+^ fluxes are mediated by the ORAI1 channel. Importantly, MAPPER-L expression reduced Mn^2+^ influx to the same extent in wild-type cells and in TKO cells expressing mCherry (mCh)-ORAI1 (25% vs 21% inhibition respectively, Fig. S7B). These data indicate that Mn^2+^ enters HEK-293 T cells predominantly via ORAI1 channels, whose activity is decreased by enforced ER tethering.

### MAPPER-L form clusters juxtaposed to STIM-ORAI complexes

**Fig. 8.**
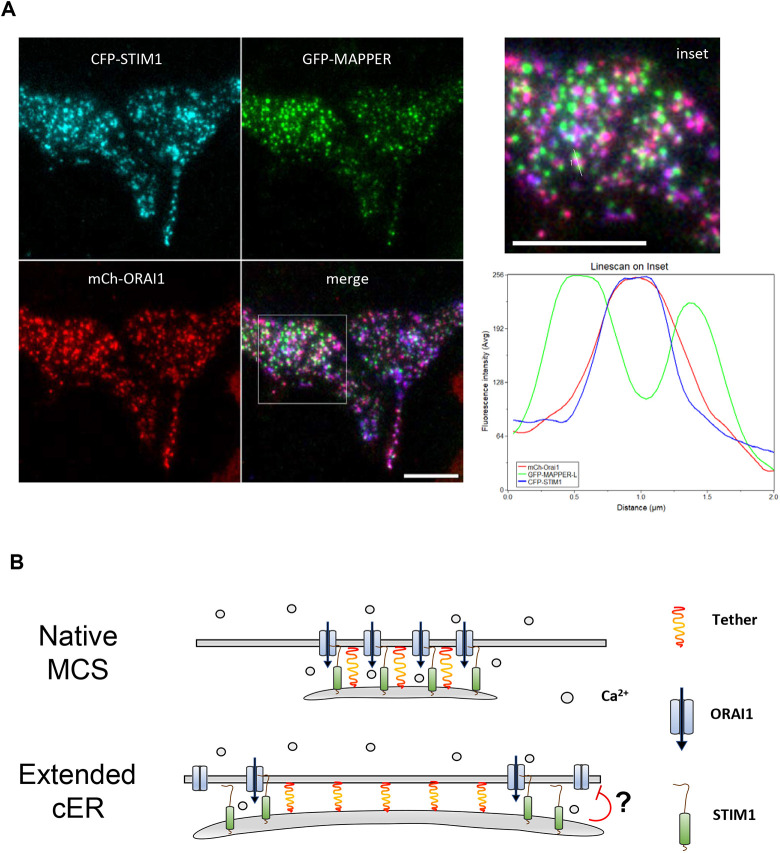
**MAPPER-L tethers cortical structures juxtaposed to STIM1/ORAI1 interaction sites.** (A) TIRF images of cells co-expressing CFP-STIM1 (cyan), mCh-ORAI1 (red), and GFP–MAPPER-L (GFP–MAPPER; green). CFP-STIM1 and mCh-ORAI1 co-localize in clusters (appearing in purple on the merged image and inset) juxtaposed to GFP–MAPPER-L clusters. The graph shows the fluorescence intensity profile along a line drawn across 3 juxtaposed clusters (shown in the inset) (cells shown are representative of 53 cells from two experiments). Scale bars: 10 µm. (B) Proposed molecular model illustrating the cER location of STIM1, ORAI1, and tether proteins during SOCE. Top, native cER expands as STIM1 proteins are recruited during store depletion. STIM1-ORAI1 complexes form along the length of cER sheets which remain elongated during Ca^2+^ entry. Bottom, enforced tether expression confines STIM1-ORAI1 complexes to the rims of cER sheets, promoting their elongation and the Ca^2+^-dependent inactivation of ORAI1 channels by accessory proteins.

We next determined the localization of the ER tethers relative to STIM-ORAI complexes by TIRF imaging. CFP-STIM1 and mCh-ORAI1 colocalized extensively while GFP–MAPPER-L formed adjacent clusters (Fig. 8A and Fig. S8A) without altering the localization and morphometric features of mCh-ORAI1 clusters (Fig. S8B). MAPPER-L thus accumulates in cER domains adjacent to STIM-ORAI interaction sites. To test whether this localization depends on the length of the tether, we expressed the SNARE protein Sec22b and its extended version Sec22b-P33 to anchor the ER at 20 and 30 nm from the PM, respectively (Petkovic et al., 2014). In sharp contrast to MAPPER-L, the two long tethers colocalized with STIM1 clusters in the TIRF plane (Fig. S8C) and their overexpression did not impact SOCE (Fig. S8D). Expression of a long linker that colocalizes with STIM1 thus fails to inhibit SOCE, while the shorter MAPPERs that inhibit SOCE form clusters juxtaposed to STIM-ORAI complexes.

## DISCUSSION

In this study, we correlate the morphological and topological changes in cortical ER structures occurring during SOCE in HEK-293 T cells expressing natural or artificial ER-PM tethers with the ionic fluxes generated by interactions between endogenous signaling proteins at these membrane contact sites. We found that native cER cisternae expand during ER Ca^2+^ depletion and remain elongated during subsequent Ca^2+^ entry whereas their ER-PM gap distance remains constant in non-transfected cells. STIM1 was previously shown to recruit and to expand cER structures upon store depletion (Orci et al., 2009; Sauc et al., 2015; Wu et al., 2006). Expression of the STIM1 interactor junctate enlarges the cER and potentiated SOCE (Treves et al., 2004), while E-Syt1 expression reduces the ER-PM gap distance by up to 12 nm during the Ca^2+^ entry phase (Fernandez-Busnadiego et al., 2015; Giordano et al., 2013; Kang et al., 2019). In all these studies, the expressed proteins are likely to be the dominant tethers. Here, we show that the only apparent ultrastructural change occurring during SOCE in non-treated HEK-293 T cells is a cER elongation that is likely mediated by endogenous STIM proteins, indicating that the cER gap distance is not dynamically regulated by the activity of endogenous E-Syt1. An earlier report showed that depletion of both E-Syt1 and E-Syt2 inhibits SOCE in Jurkat T cells but not in HeLa cells (Woo et al., 2020) and our siRNAs targeting either isoform did not inhibit SOCE in HEK-293 T cells (data not shown). This might reflect the expression of distinct isoforms in immune and non-immune cells, with the activating isoform E-Syt2b possibly accounting for the inhibitory effects of E-Syt1 and E-Syt2 silencing in Jurkat T cells.

Expression of E-Syt1 or of MAPPER-S had no significant impact on cER structures in non-treated cells, but the expression of E-Syt2 and MAPPER-L induced the formation of abnormally elongated cER cisternae reaching a length of up to 900 nm. This is consistent with the potent tethering function of E-Syt2 (Fernandez-Busnadiego et al., 2015; Idevall-Hagren et al., 2015) and indicates that MAPPER-L is not an innocuous cER marker but alters its ultrastructure. This remodeling effect might be related to the very high avidity of the polybasic tail of the chimeric MAPPER proteins for phosphoinositides and indicates that MAPPER should not be used to label the cER. In an earlier cryo-ET study, large ER-PM contacts were observed in COS-7 cells overexpressing E-Syt3 and occasionally in cells overexpressing E-Syt1 (Fernandez-Busnadiego et al., 2015). These contacts resemble the elongated cER cisternae that we observed in cells overexpressing E-Syt2 and MAPPER-L, suggesting that cER expansion is a common phenomenon associated with the expression of tethering proteins.

Following Ca^2+^ stores depletion with Tg, the cER elongation was greatly potentiated by the expression of any of the four tether proteins. The strongest effects were observed with E-Syt2 and MAPPER-L, in line with their effects in non-treated cells. Interestingly, the ER-PM gap distance stabilized at a fixed distance specific for each tether family as the cER cisternae elongated, around 12-15 nm for the E-Syts and 5-9 nm for the MAPPERs. This pattern contrasts sharply with the variability of the gap distances observed in smaller (<300 nm) cER cisternae, which ranged from 5 to 30 nm regardless of tether expression. Another unexpected finding was that the cER elongation associated with tether expression decreased during the Ca^2+^ influx phase whereas the ER-PM gap distances remained constant. E-Syt1 expression in HEK-293 T cells was previously reported to reduce the ER-PM gap distance by 12 nm upon Ca^2+^ entry, as estimated from changes in fluorescence intensity in variable-angle TIRF imaging (Kang et al., 2019). The increased fluorescence intensity reported in this study might reflect the increased concentration of fluorescent molecules in contracting cER structures remaining anchored at a fixed distance of 12-15 nm to the PM.

The average ER-PM distance of native contacts was 25.4 nm in a cryo-electron tomography study of COS-7 cells (Fernandez-Busnadiego et al., 2015), which is significantly higher than the averaged gap distance of 16.8 nm that we report by scoring >100 contacts in Epon-embedded HEK-293 T cells. This difference might reflect differences between cell types or in the EM procedures, as the osmium labeling of membranes and embedding in resin might reduce the apparent gap distance in our 30 nm-thick slices. In COS-7 cells overexpressing E-Syt1, the averaged gap distance in cryo-ET was 21.8 nm and decreased to 14.8 nm following Tg-induced SOCE with 2 mM external Ca^2+^ (Fernandez-Busnadiego et al., 2015). In our HEK-293 T cells expressing E-Syt1, the ER-PM gap decreased from 17.3 to 14.9 nm during Tg addition and to 12.8 nm if considering only the longest cER structures. Thus, the gap distance was reduced at an earlier step during the SOCE protocol, suggesting that the Ca^2+^ elevation generated by the release of Ca^2+^ from ER stores during Tg application is sufficient to recruit E-Syt1 to the PM and to induce a shortening of the ER-PM gap distance. This is consistent with the reported Ca^2+^-dependent tethering function of E-Syt1 (Fernandez-Busnadiego et al., 2015; Giordano et al., 2013).

Counterintuitively, the cER expansion imparted by the expressed tether did not enhance SOCE but instead reduced the incoming fluxes of ions generated by STIM-ORAI interactions at MCSs. In cells expressing E-Syt2 and MAPPER-L, which caused maximal cER expansion, the rates of Ca^2+^ influx evoked by the Tg-readmission protocol were reduced by ∼50%. The effect was observed in several cell types and persisted when the Ca^2+^ concentration was reduced to 0.5 mM but disappeared at 10 mM Ca^2+^, the highest concentration tested. MAPPER-S expression also reduced SOCE in HeLa cells or when the reversible SERCA inhibitor CPA was used to evoke SOCE. CPA enables store refilling, which would further reduce the cytosolic Ca^2+^ concentration during the influx phase. These data thus indicate that the expression of tethering proteins that promote cER expansion reduce SOCE, and that this effect is preferentially detected at low cytosolic Ca^2+^ concentrations with the depletion-readmission protocol. The cER expansion also impacted the Ca^2+^ homeostasis of intracellular Ca^2+^ stores, reducing the amount of Ca^2+^ mobilized by an InsP3-mediated agonist. This defect was not associated with a reduction of the amount of Ca^2+^ released by Tg, suggesting that it could reflect a loss of active InsP3 receptors near sites of store-operated Ca^2+^ entry (Thillaiappan et al., 2021). The activity of the PMCA remained unaffected and Ca^2+^ influx remained sensitive to the expression of CaM mutants, indicating that the regulation of STIM-ORAI complexes by accessory proteins is conserved. Using Mn^2+^, we could quantify the effect of ER tethering on cationic fluxes recorded before and after store depletion and Ca^2+^ readmission, and correlate these PM fluxes with the cER size measured in the same experimental conditions. These experiments revealed that the expression of MAPPERs reduces Mn^2+^ entry marginally after store depletion and maximally at the peak of the cytosolic Ca^2+^ elevation. These data indicate that enforced cER elongation limits SOCE, suggesting that part of the elongated cER structures do not favor STIM-ORAI interactions. Accordingly, STIM-ORAI clusters were juxtaposed with MAPPER-L in the TIRF plane (Fig. 8A).

Based on our morphological observations, we propose that during STIM1 recruitment to the PM, the cER expands as new interactions are formed between incoming STIM1 proteins and phospholipids. In the presence of an excessive amount of tethers, the newly formed STIM1-PM interactions are replaced by more stable interactions between the PM and the expressed tethers, which have a higher affinity for phospholipids (Heo et al., 2006), thereby promoting cER expansion and stabilization at a fixed distance (Fig. 8). Upon Ca^2+^ elevations, the electrostatic interactions between negatively charged phospholipids and the tethers might be weakened, reversing the elongation process, while the interactions between STIM and ORAI proteins are disrupted by Ca^2+^-bound CaM and by other Ca^2+^-dependent negative regulators like SARAF (Fig. 8). Our functional observations further suggest that the artificially elongated cER cisternae do not allow productive STIM-ORAI interactions in their center since the cER length was not correlated with the SOCE amplitude. This suggests that the elongated cERs are populated predominantly by the exogenous tethers, with STIM-ORAI complexes restricted to the periphery of the MCSs, consistent with an earlier report that STIM1-ORAI1 complexes accumulate at the periphery of cER cisternae artificially tethered 4–6 nm from the PM (Varnai et al., 2007). Alternatively, STIM proteins might populate the expanded structures but revert to a globular, resting conformation, thereby reducing the amount of extended STIM1 proteins available for gating. This would be consistent with our previous observation that overexpressed YFP-STIM is concentrated in thin regions of the cER close to the PM (Orci et al., 2009).

In summary, we show that cER sheets expand during store depletion and that this elongation persists during subsequent Ca^2+^ elevations. Enforced expression of ER-PM tethers maximizes cER expansion and fixes the enlarged cisternae at specific gap distances in a process partially reversed by Ca^2+^ elevations. cER enlargement negatively regulates Ca^2+^ release and SOCE, likely by sequestering STIM-ORAI complexes, and possibly InsP3 receptors, at the periphery of the enlarged cER sheets. These findings indicate that ER-PM tethering negatively regulates SOCE by promoting cER expansion, a remodeling that might participate in the termination of store-operated Ca^2+^ entry.

## MATERIALS AND METHODS

### Reagents

The following reagents were used in this article; thapsigargin (67526-95-8, Sigma), cyclopiazonic acid from *Penicillium cyclopium* (18172-33-3, Sigma), Mg-ATP (Sigma), MnCl_2_ (13446-34-9, Sigma), Fura-2-AM (FP-42776A, Interchim), Deep Red CellMask (C10046, Thermo Fisher Scientific) and Lipofectamine 2000 (11668019, Thermo Fisher Scientific). Ca^2+^ recording buffers contained: 140 mM NaCl, 5 mM KCl, 1 nM MgCl_2_, 20 mM HEPES, 10 mM glucose, supplemented with 1 mM EGTA or 0.5-10 mM CaCl_2_ as indicated.

### Plasmids

pEGFP-C1 was purchased from ClonTech (#6084_1); pcDNA-D1_ER_ (#36325), EGFP-E-Syt1 (#66830), EGFP-E-Syt2 (#66831) and pCMV-R-CEPIA1er (#58216) were from Addgene. MAPPER constructs were a kind gift from Jen Liou, UT Southwestern, Texas; CaM constructs (pCDNA3–CFP–hCaM and pCDNA3–CFP–hCaM1-4-mutant, Griessmeier et al., 2009) were gifts from Rajesh Bhardwaj, University of Bern, Switzerland.

### Cell culture and transfections

Human embryonic kidney (HEK-293 T) cells were obtained from ATCC (CRL-11268, Manassas, VA, USA) maintained in Dulbecco's modified Eagle's medium (DMEM, Thermo Fisher Scientific, cat. no. 31966-021) supplemented with 10% fetal bovine serum and 1% penicillin-streptomycin, grown at 37°C and 5% CO_2_ and regularly tested for mycoplasma. MEF cells (kindly provided by Luca Scorrano, University of Padova, Italy) were grown in identical conditions. HeLa cells purchased from the European Collection of Authenticated Cell Cultures (ECACC) were grown in MEM Gibco (41090). Cell lines used in this study tested negative for mycoplasma contamination, which was performed on a monthly basis. HEK 293 T and HeLa cells were genetically confirmed (by genomic profiling [STRs]) prior to expansion and stockage. Prior to experiments, cells were seeded on coverslips of 25 mm coated with poly-L-lysine (P4832, Sigma-Aldrich) and transfected 1-day post-seeding using 200 ng of plasmids for HEK-293 T, 0.5 µg for MEF and 1 µg for HeLa cells mixed with 3 µl Lipofectamine 2000. The DNA-Lipofectamine mix was added to cells for 4 h and the culture medium was replaced.

### Transmission electron microscopy

Cells were sorted by flow cytometry (MoFlo Astrios, Beckman) for similar GFP fluorescence levels 24 h post transfection and re-seeded for 24 h on plastic dishes coated with poly-L-lysine. Cells were left untreated (NT), exposed to 1 µM (Tg) for 10 min in the absence of Ca^2+^ (CF) or treated with Tg as above and re-exposed to 1 mM Ca^2+^ for 2 min (CA), fixed with 2.5% glutaraldehyde, stained with uranyl acetate, postfixed with osmium tetroxide, and embedded in Epon resin. After sectioning at 30 nm, samples were imaged on a Technai 20 transmission electron microscope (FEI, Eindhoven, Netherlands) at 92,000 times magnification. For each cell identified on the grid every ER structure located within 30 nm of the PM was imaged. The cER length and the ER-PM gap distance were determined on EM images using ImageJ/Fiji and MATLAB, respectively. A segmented line was drawn in ImageJ/Fiji across the cER long axis to extract the cER length. The ER-PM gap distances was obtained from two lines drawn on the PM and proximal cER membranes whose pixel-to-pixel geodesic distances were extracted and averaged for each cER. Only structures located at less than 30 nm from the PM were analyzed.

### Ca^2+^ imaging

Ca^2+^ imaging was performed as described previously (Sauc et al., 2015). Briefly, cells were loaded with 4 µM Fura-2-AM in CA medium for 25 min at room temperature (25°C) before imaging on a Nikon Eclipse Ti microscope (Nikon Instruments), equipped with a Lambda DG4 illumination system (Sutter Instrument) and a 16-bit CMOS camera (pco.Edge sCMOS, Visitron Systems). Cells were selected for similar GFP fluorescence and the following filter sets were used (all from Chroma Technology Corp.): for Fura-2 ratio recordings, ET340x-ET380x-ET510/80m-T400lp; for Mn^2+^ quench, ET365/10x; and for R-CEPIA1_ER_ recordings, ET572/35x-69002bs-ET630/75 m. We piloted the setup with Visiview software (Visitron) using a Python script to better control the time resolution during the protocol (https://github.com/Carandoom/VisiviewFura2). Ca^2+^ images were analyzed using an ImageJ script (https://github.com/Carandoom/Fura2Analysis; https://zenodo.org/badge/latestdoi/373477639). Using regions drawn on the background and on each cell of interest, the script removes the mean background fluorescence and extracts the mean fluorescence of each cell region for each frame to calculate the fluorescence ratio and graph it as R/R0 in Excel. The rates of Ca^2+^ entry were calculated with a MATLAB script (https://github.com/Carandoom/SlopeFromLinearRegression; https://zenodo.org/badge/latestdoi/373492736). Briefly, the script plots the recording, allowing the user to analyze a positive or negative slope within a given range of timepoints. The script calculates the first derivative to define a region to fit a linear regression and extracts a slope parameter.

### TIRF microscopy

TIRF imaging was performed on a Nikon Eclipse Ti microscope equipped with a Perfect Focus System (PFS III) and a 100× oil CFI Apochromat TIRF Objective (NA 1.49; Nikon Europe B.V.). For 488 nm excitation, the filter cube contained a ZET488/10 excitation filter (Chroma), a 502 nm dichroic mirror (H 488 LPXR superflat) and a 530/43 Bright Line HC emission filter (Semrock, Inc.). For CellMask imaging, we used a 640 nm laser and Cy5 700/75 emission filter. The fluorescence was collected by an EMCCD camera cooled at −80°C (iXon Ultra 897, Andor Technology Ltd) piloted with NIS-Elements Ar software V4.13 (Nikon). The PM was labeled with deep red CellMask (1:10,000 in CA for 10 min at room temperature) and used to adjust the TIRF angle. Fluorescence puncta on TIRF images were segmented using a custom ImageJ/Fiji script available on GitHub (https://github.com/Carandoom/STIM-ORAI-Segmentation; https://zenodo.org/badge/latestdoi/373828941).

### Data analysis

Statistical analyses were performed with GraphPad Prism 9, *P* values are labeled *P≤0.05, **P≤0.01, ***P≤0.001 and ****P≤0.0001. All tests conducted were two-tailed unless otherwise indicated in the figure legends. The data that support the findings of this study are present in the supplementary figures, with number of experiments and statistical tests applied. Data size is defined by cells (*n*) within a minimum of three biologically independent experiments, considered as an independent transfection recorded on a different day. Samples allocation/randomisation were not pre-defined for this article.

## Supplementary Material

Supplementary information

Reviewer comments
