# Supplementary material for: Enforced tethering elongates the cortical endoplasmic reticulum and limits store-operated Ca2+ entry
Source: J Cell Sci. 2022 Mar 30;135(6):jcs259313. doi: 10.1242/jcs.259313 (PMC8995094; doi:10.1242/jcs.259313)
Supplement: Supplementary information [file joces-135-259313-s1.pdf]

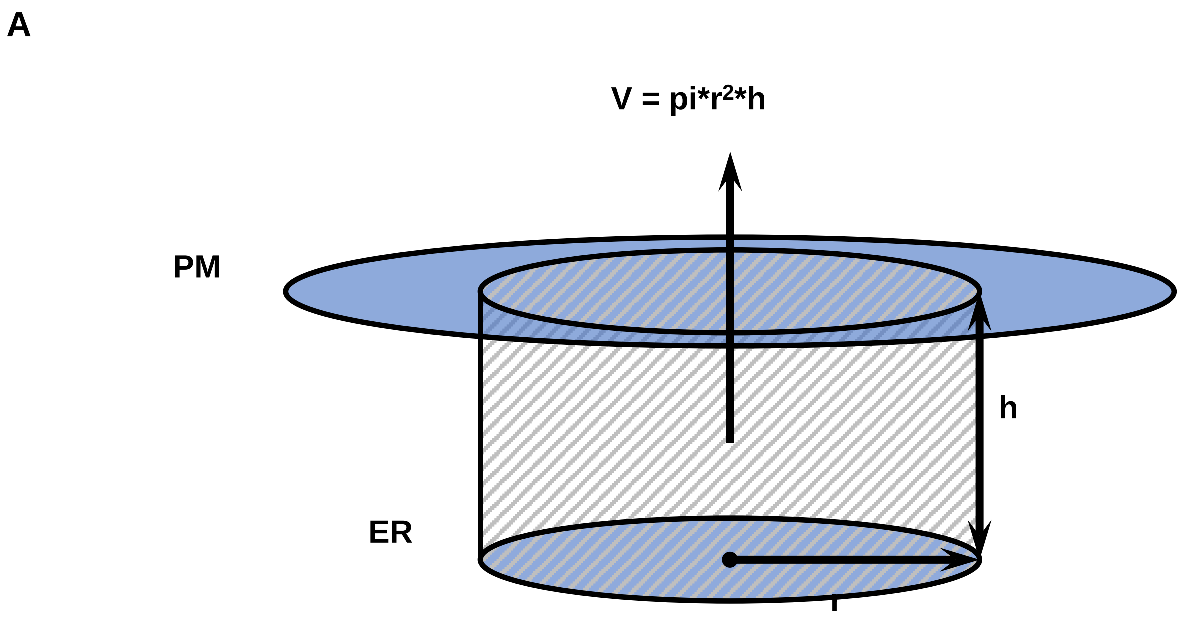

Fig. S1. Calculation of ER-PM cleft volume.
Cleft volume was calculated using cER length and gap values assuming a cylindric shape. V: Volume; r: radius; h; height.

Journal of Cell Science • Supplementary information

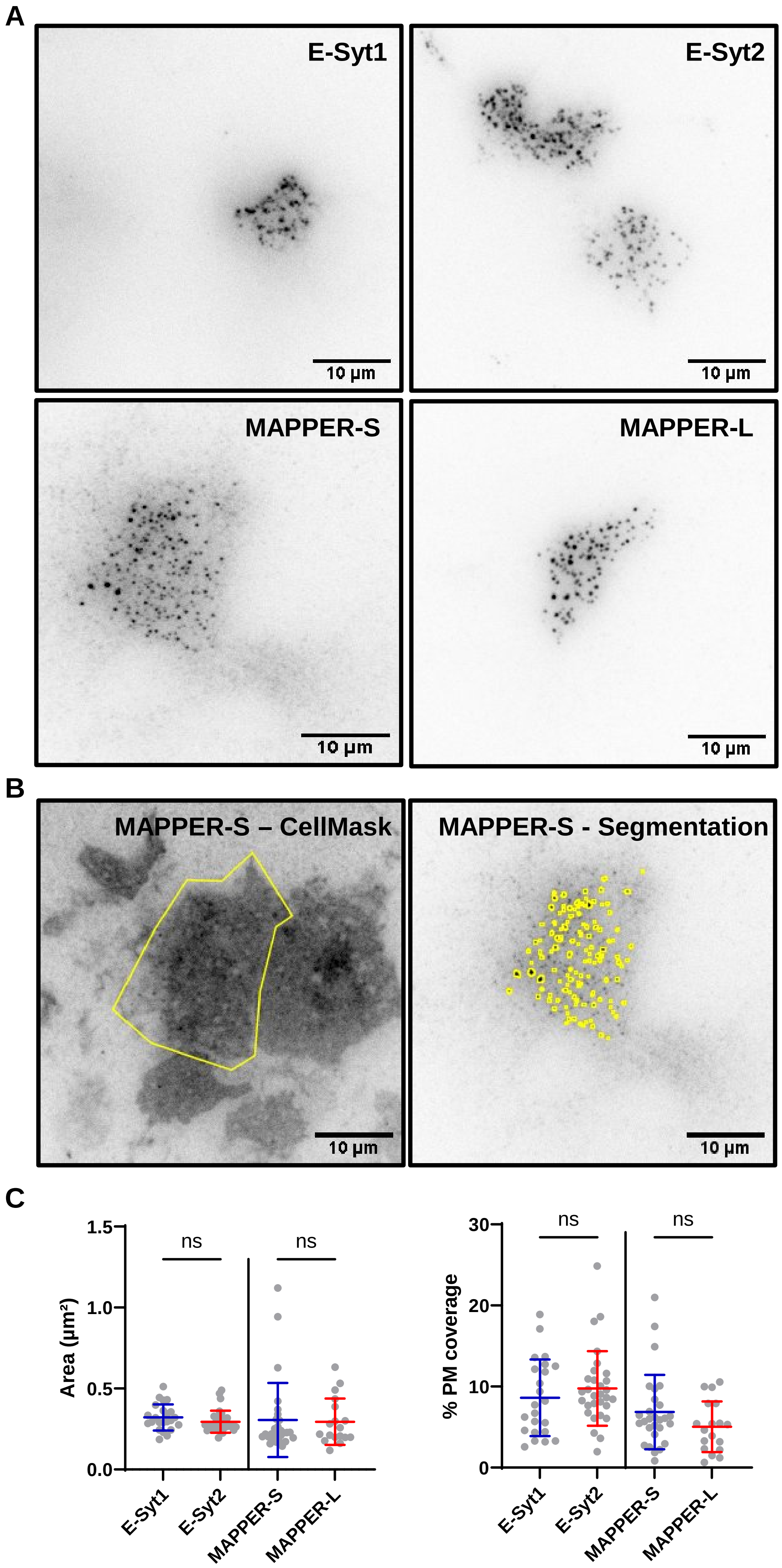

Journal of Cell Science • Supplementary information

Fig. S2. E-Syts and MAPPERs populate similar near-PM clusters following store depletion. A) Representative TIRF images of HEK-293T cells expressing the indicated GFP-tagged tether proteins. Cell membrane was labelled with CellMask, treated 10 min with 1 µM Tg in Ca^{2+}-free, and fixed. B) CellMask image of MAPPER-S-expressing cells from A, with the cell border (left) and GFP clusters (right) outlined in yellow. C) Quantification of the cluster area (left) and membrane coverage (right) in cells expressing the different tethers. Data are mean ±SEM of (E-Syt1 = 18, E-Syt2 = 30, M-S = 28, M-L = 18 cells) ns: not significant, one-way ANOVA.

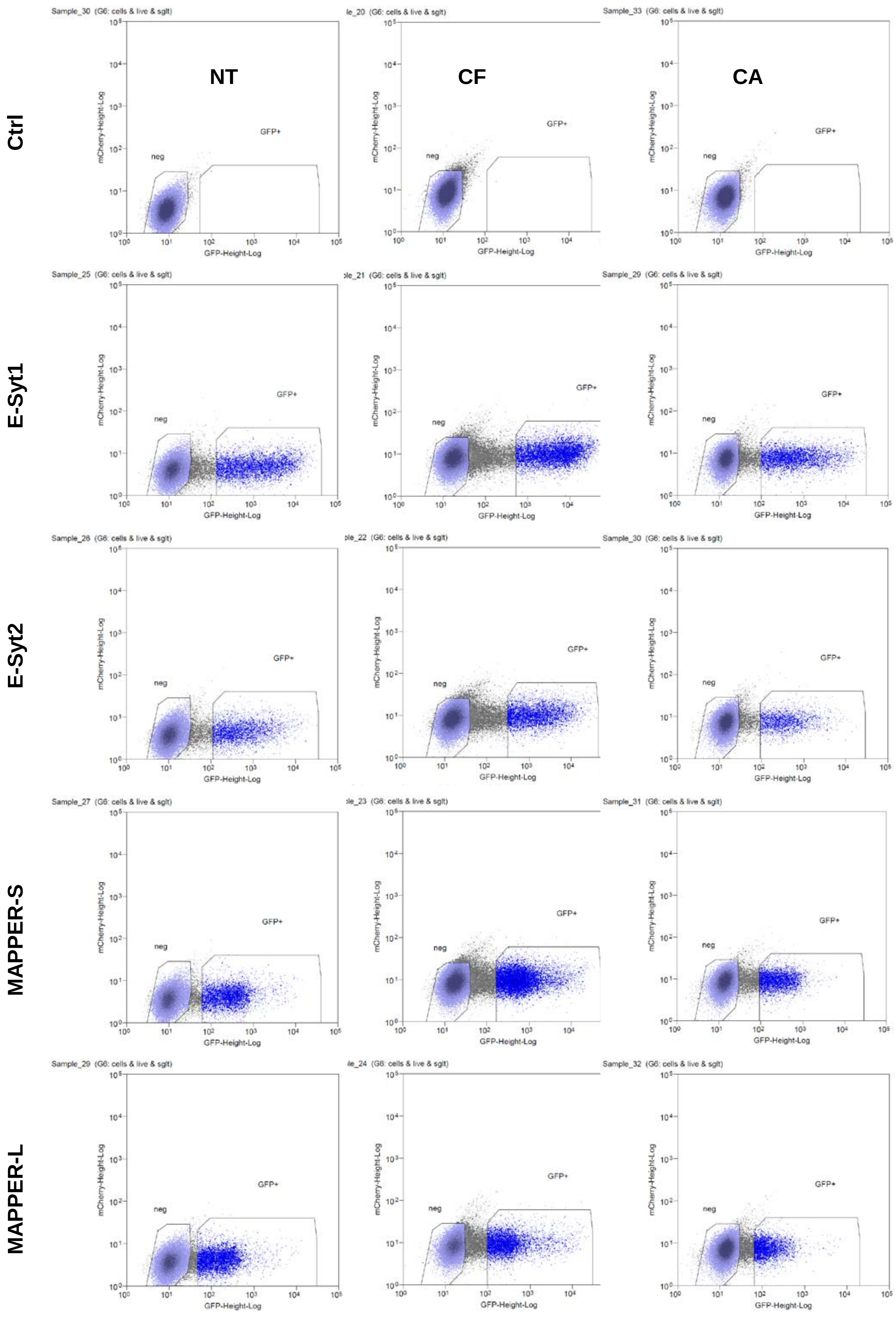

Journal of Cell Science • Supplementary information

Fig. S3. Sorting strategy of cells expressing ER-PM tether proteins.
Cells expressing GFP-tagged E-Syts and MAPPERs were sorted by flow cytometry for similar GFP fluorescence in NT, CF, and CA conditions.

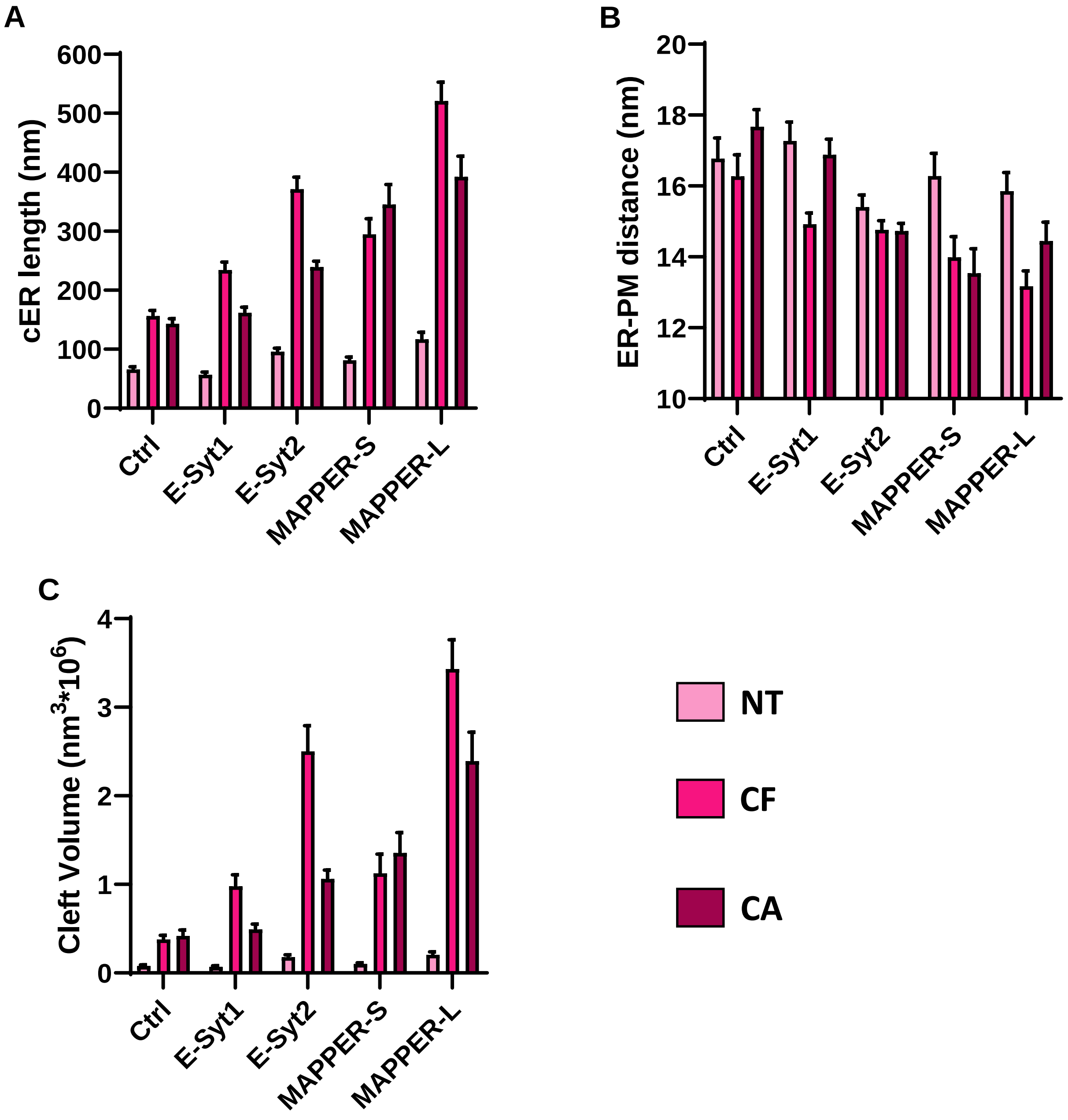

Fig. S4. E-Syts and MAPPERs expression augments the cER elongation during SOCE.
A-C) Quantification of the averaged ER-PM gap distance (A), cER length (B) and cleft volume (C) in cells expressing E-Syts and MAPPERs and imaged in NT, CF, and CA conditions. Data are mean±SEM. N numbers are presented on Table and 2.

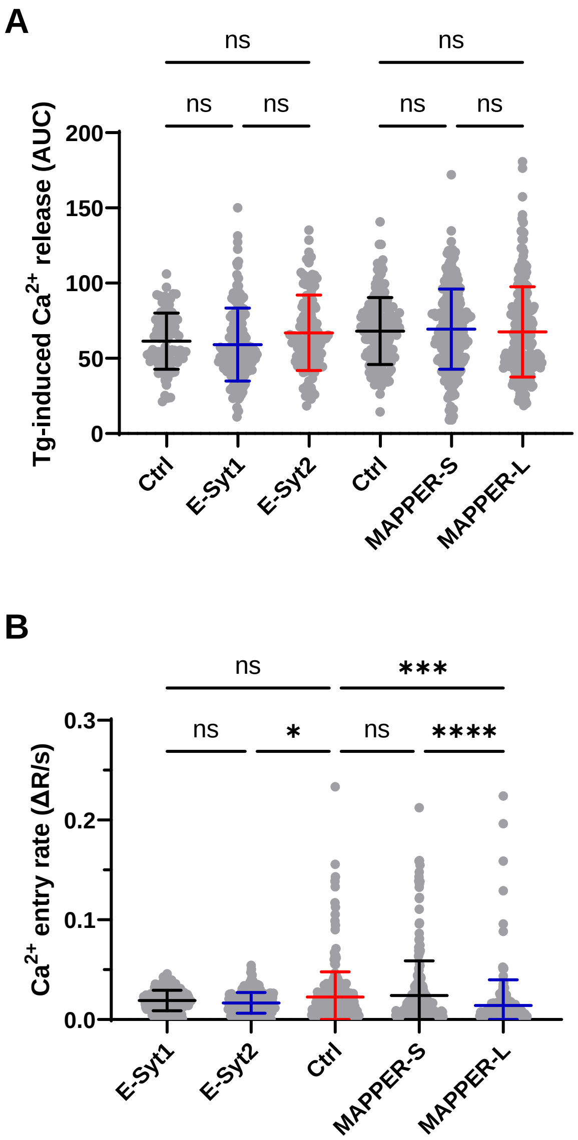

Fig. S5. E-Syt2 and M APPER-L expression inhibit SOCE but not Ca^{2+} release

A) Quantification of the amount of Ca^{2+} released by Tg in Ca^{2+}-free medium in cells expressing E-Syts and MAPPERs. Data are mean±SEM of; left to right n = 83, 154, 126, 248, 226 cells. B) Quantification of Ca^{2+} entry rate in cells expressing GFP-tagged E-Syts and MAPPERs, sorted by flow cytometry for similar GFP fluorescence. Data are mean±SEM of ; left to right n = 321, 238, 242, 256, 225 cells *p<0.05, ***p<0.001, ****p<0.0001, one-way ANOVA.

Journal of Cell Science • Supplementary information

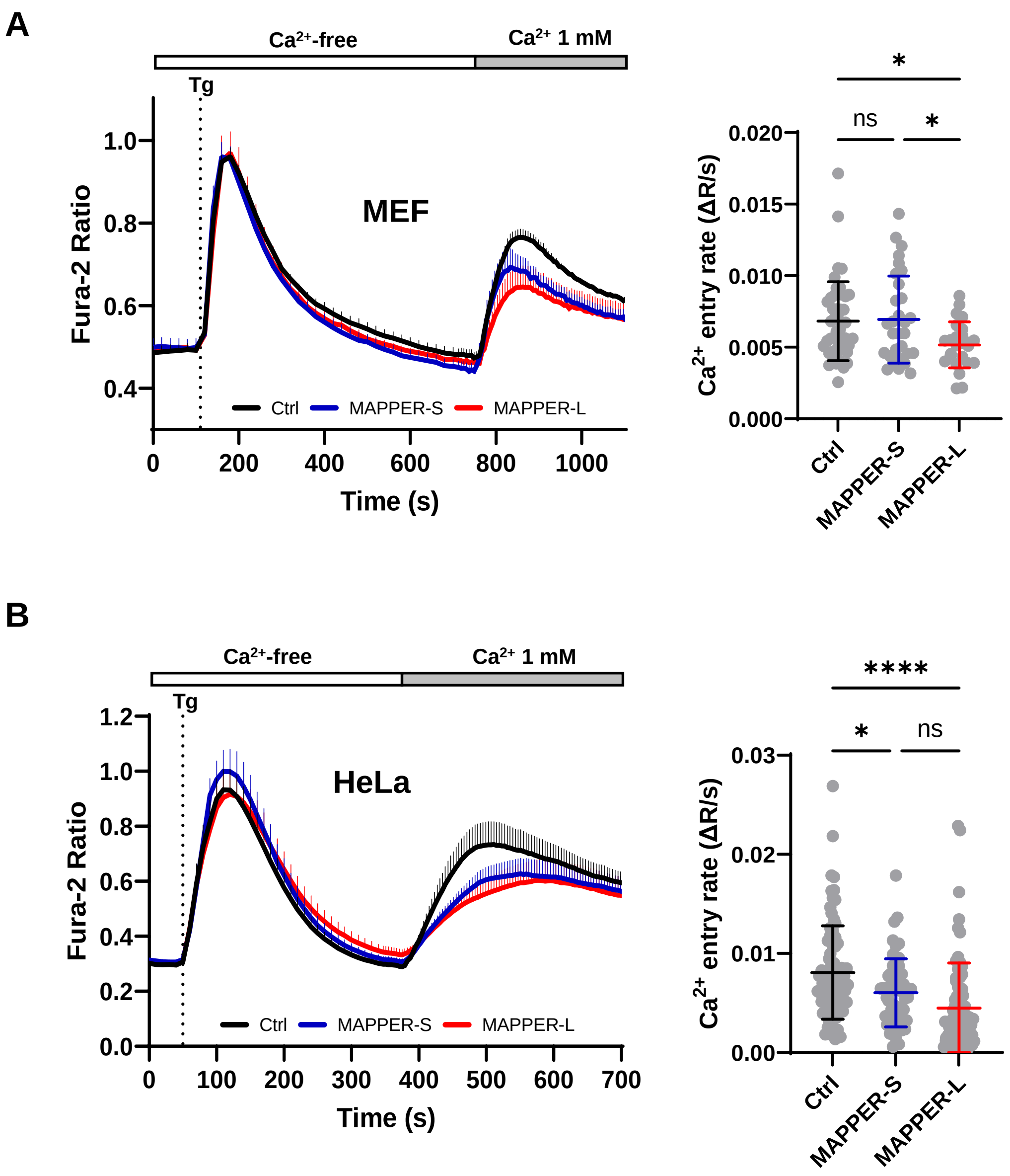

Fig. S6. Effect of MAPPER expression in MEF and HeLa cells

Averaged Ca2+ recordings (left) and Ca^{2+} extrusion rates (right) in MEF (A) MAPPER-S (n = 32), MAPPER-L (n = 25) or a control plasmid (n = 47) and HeLa cells (B) expressing MAPPER-S (n = 55), MAPPER-L (n = 75) or a control plasmid (n = 83). *p<0.05, ***p<0.001, ****p<0.0001, one-way ANOVA.

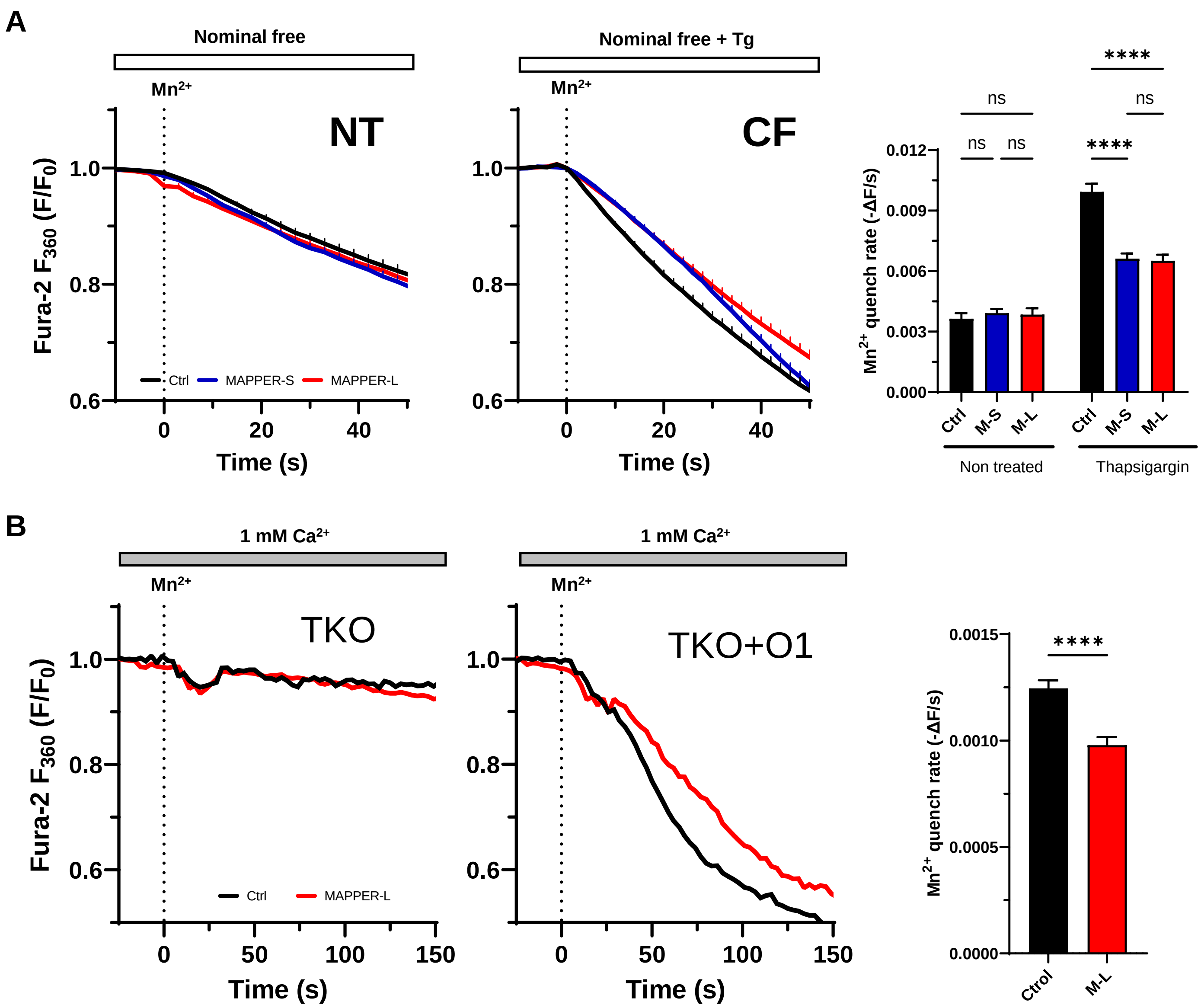

Fig. S7. MAPPERs expression inhibits store-operated Mn^{2+} entry via Orai1.
A. Averaged Mn^{2+} quench recordings in cells expressing MAPPER-S (n = 167), MAPPER-L (n = 169) or a control plasmid (n = 117) before stimulation (Left, NT) and after store depletion (M-S = 193; M-L = 202; Ctrl = 179) (Middle, CF). Recordings were in nominal Ca^{2+}-free medium without EGTA to avoid Mn^{2+} chelation. Right: Statistical evaluation of the Mn^{2+} quenching rates. MAPPERs expression strongly reduced Mn^{2+} entry after Ca^{2+} readmission. Data are mean±SEM of cells from 5-12 independent recordings. *p<0.05, ***p<0.001, ****p<0.0001, one-way ANOVA. B. Representative Mn^{2+} quench recordings of ORAI1-3-deficient HEK-293 cells (Triple KO, TKO) expressing the indicated constructs either alone (left) or together with mCh-ORAI1 (middle). Recordings were in 1 mM Ca^{2+} readded for 2 min following Tg exposure for 10 min in Ca^{2+}-free solution. Right: Statistical evaluation of Mn^{2+} quench rates. Data are mean±SEM of 91 (Ctrl) and 66 (M-L) cells from 5-6 independent recordings, ****p<0.0001, Student t test.

Journal of Cell Science • Supplementary information

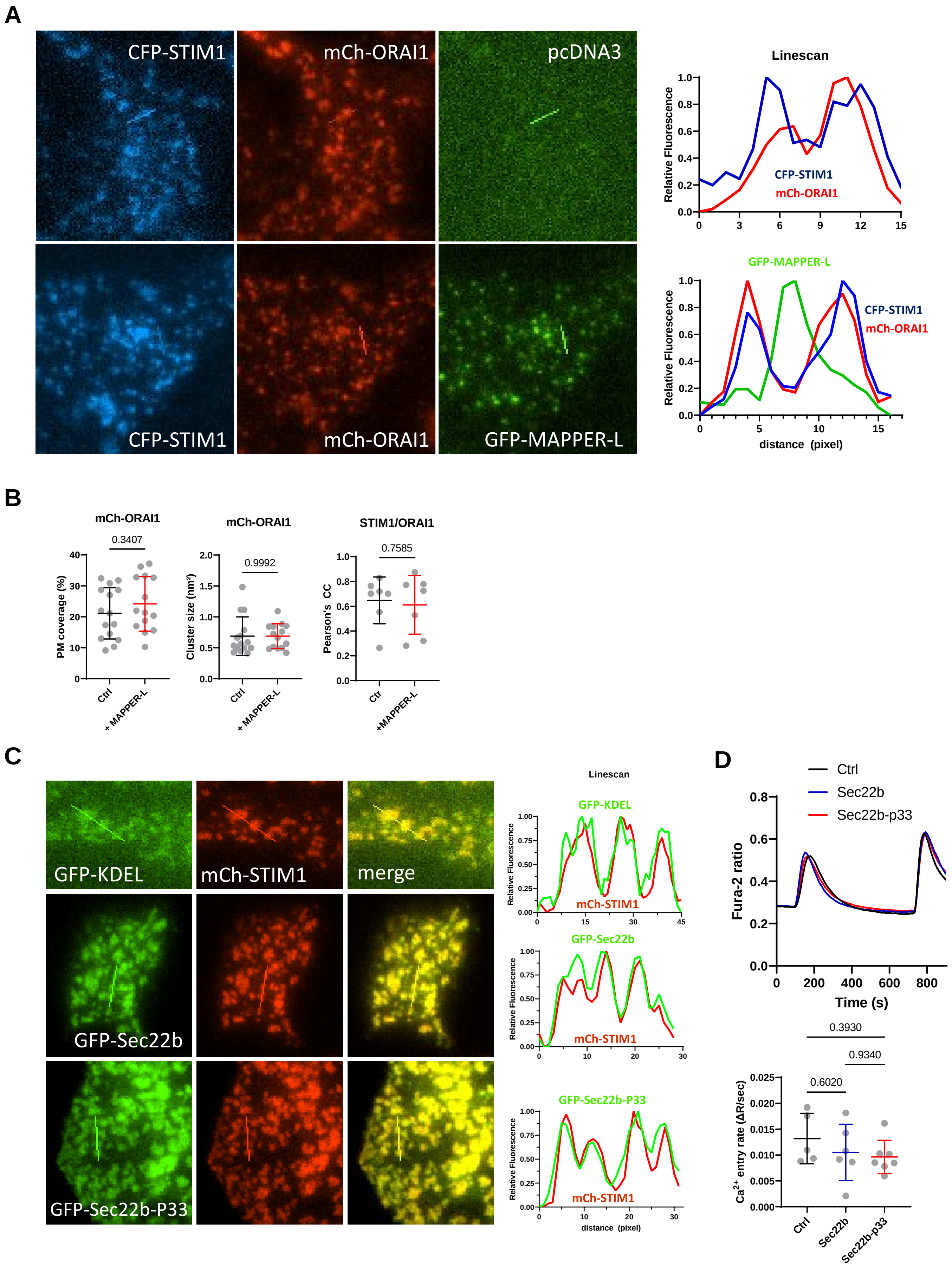

Journal of Cell Science • Supplementary information

Fig. S8. MAPPER-L tethers cortical structures juxtaposed to STIM/ORAI interaction sites.
A. TIRF images of cells co-expressing CFP-STIM1 (cyan) and mCh-Orai1 (red) without (top images) or with (bottom images) GFP-MAPPER-L (green). MAPPER-L expression did not alter the localization of STIM/ORAI clusters. Graphs shows the fluorescence intensity profile along a line (shown on images) drawn across STIM/ORAI and juxtaposed MAPPER-L clusters. Bars: 10 µm. B) Morphometric parameters of mCh-ORAI1 clusters in cells expressing or not GFP-MAPPER-L (n = 15 and 14 cells respectively). The fraction of the PM decorated (left), the area of individual clusters (middle), and the Pearson's co-localization coefficients were not altered by the expression of GFP-MAPPER-L. C. TIRF images of cells co-expressing mCh-STIM1 (red) and the indicated GFP-tagged constructs (green). Graphs shows the fluorescence intensity profile along a line (shown on images) drawn across mCh-STIM1 clusters. The ER marker GFP-KDEL, the SNARE protein Sec22b, and its extended version Sec22b-P33 colocalized with mCh-STIM1 clusters. Bars: 10 µm. D. Top: Averaged fura-2 recordings of Ca^{2+} elevations evoked by the Tg-readmission protocol in cells expressing Sec22b, Sec22b-P33, or a control plasmid. Bottom: Quantification of Ca^{2+} entry rates. Averaged slope from 5 (ctrl) 6 (Sec22b) and 7 (p33) independent experiments.

Table S1. Quantification of cortical ER parameters

Epon sections were generated before stimulation (NT), after store depletion (CF), and following Ca^{2+} readmission (CA). The total number of contact sites measured is indicated for each condition.

		ER-PM distance (nm)		cER length (nm)		
	**Condition**	**Mean**	**SD**	**Mean**	**SD**	**N**
**Ctrl**	**NT**	16,77	6,02	65,8	45,5	105
	**CF**	16,27	5,77	156,0	92,4	89
	**CA**	17,67	5,85	143,1	106,5	146
**E-Syt1**	**NT**	17,27	5,33	56,8	42,3	100
	**CF**	14,92	4,78	234,2	206,2	226
	**CA**	16,88	5,38	161,9	115,7	149
**E-Syt2**	**NT**	15,40	4,50	95,7	79,5	167
	**CF**	14,76	3,68	371,1	290,2	194
	**CA**	14,73	4,25	239,3	198,1	402
**MAPPER-S**	**NT**	16,28	6,70	81,4	57,6	110
	**CF**	13,99	6,74	294,5	309,2	133
	**CA**	13,54	7,74	345,3	384,6	127
**MAPPER-L**	**NT**	15,85	6,97	116,5	161,7	175
	**CF**	13,17	6,52	520,5	485,2	223
	**CA**	14,45	6,73	392,0	447,5	162

NT: Non treated
CF: EGTA 1 mM + Tg 10 min
CA: EGTA 1 mM + Tg 10 min then Ca^{2+} 1 mM for 2 min

Table S2. Statistical evaluation of cER morphological parameters.
Cells expressing E-Syts and MAPPERs were imaged in NT, CF, and CA conditions. *p<0.05, **p<0.01, ***p<0.001, ****p<0.0001, one-way ANOVA.

		ER-PM distance		MCS length	
	Tukey's multiple comparisons test	Summary	Adjusted P Value	Summary	Adjusted P Value
Ctrl	NT vs. CF	ns	0.8148	*	0.0421
	NT vs. CA	ns	0.432	ns	0.0522
	CF vs. CA	ns	0.1602	ns	0.9275
E-Syt1	NT vs. CF	**	0.0017	****	<0,0001
	NT vs. CA	ns	0.8565	**	0.005
	CF vs. CA	**	0.0032	*	0.0228
E-Syt2	NT vs. CF	ns	0.5357	****	<0,0001
	NT vs. CA	ns	0.4073	****	<0,0001
	CF vs. CA	ns	0.998	****	<0,0001
MAPPER-S	NT vs. CF	**	0.0052	****	<0,0001
	NT vs. CA	***	0.0007	****	<0,0001
	CF vs. CA	ns	0.7995	ns	0.2558
MAPPER-L	NT vs. CF	****	<0,0001	****	<0,0001
	NT vs. CA	ns	0.0623	****	<0,0001
	CF vs. CA	ns	0.075	****	<0,0001
NT	Ctrl vs. E-Syt1	ns	0.9705	ns	0.9992
	Ctrl vs. E-Syt2	ns	0.3000	ns	0.8876
	E-Syt1 vs. E-Syt2	ns	0.0709	ns	0.7613
	Ctrl vs. MAPPER-S	ns	0.9701	ns	0.9921
	Ctrl vs. MAPPER-L	ns	0.6852	ns	0.5083
	MAPPER-S vs. MAPPER-L	ns	0.9718	ns	0.8010
CF	Ctrl vs. E-Syt1	ns	0.3199	ns	0.1137
	Ctrl vs. E-Syt2	ns	0.2323	****	<0,0001
	E-Syt1 vs. E-Syt2	ns	0.9985	****	<0,0001
	Ctrl vs. MAPPER-S	*	0.0286	***	0.0010
	Ctrl vs. MAPPER-L	***	0.0001	****	<0,0001
	MAPPER-S vs. MAPPER-L	ns	0.6818	****	<0,0001
CA	Ctrl vs. E-Syt1	ns	0.7560	ns	0.9716
	Ctrl vs. E-Syt2	****	<0,0001	**	0.0012
	E-Syt1 vs. E-Syt2	***	0.0008	*	0.0163
	Ctrl vs. MAPPER-S	****	<0,0001	****	<0,0001
	Ctrl vs. MAPPER-L	****	<0,0001	****	<0,0001
	MAPPER-S vs. MAPPER-L	ns	0.6603	ns	0.5510

NT: Non treated
CF: EGTA 1 mM + Tg 10 min
CA: EGTA 1 mM + Tg 10 min then Ca^{2+} 1 mM for 2 min
