# Supplementary material for: Enforced tethering elongates the cortical endoplasmic reticulum and limits store-operated Ca2+ entry
Source: J Cell Sci. 2022 Mar 30;135(6):jcs259313. doi: 10.1242/jcs.259313 (PMC8995094; doi:10.1242/jcs.259313)
Supplement: Reviewer comments [file joces-135-259313_review_history.pdf]

Journal of Cell Science

Enforced tethering elongates the cortical endoplasmic reticulum and limits store-operated Ca^{2+} entry

Christopher Henry, Amado Carreras-Sureda and Nicolas Demaurex
DOI: 10.1242/jcs.259313

Editor: Jennifer Lippincott-Schwartz

Review timeline

Original submission:	30 August 2021
Editorial decision:	27 October 2021
First revision received:	31 January 2022
Accepted:	14 February 2022

Original submission

First decision letter

MS ID#: JOCES/2021/259313

MS TITLE: Enforced tethering elongates the cortical endoplasmic reticulum and limits store-operated calcium entry

AUTHORS: Christopher Henry, Amado Carreras Sureda, and Nicolas Demaurex
ARTICLE TYPE: Research Article

We have now reached a decision on the above manuscript.

To see the reviewers' reports and a copy of this decision letter, please go to: https://submit-jcs.biologists.org and click on the 'Manuscripts with Decisions' queue in the Author Area. (Corresponding author only has access to reviews.)

As you will see, the reviewers raise a number of substantial criticisms that prevent me from accepting the paper at this stage. They suggest, however, that a revised version might prove acceptable, if you can address their concerns. If you think that you can deal satisfactorily with the criticisms on revision, I would be pleased to see a revised manuscript. We would then return it to the reviewers.

We are aware that you may be experiencing disruption to the normal running of your lab that makes experimental revisions challenging. If it would be helpful, we encourage you to contact us to discuss your revision in greater detail. Please send us a point-by-point response indicating where you are able to address concerns raised (either experimentally or by changes to the text) and where you will not be able to do so within the normal timeframe of a revision. We will then provide further guidance. Please also note that we are happy to extend revision timeframes as necessary.

Please ensure that you clearly highlight all changes made in the revised manuscript. Please avoid using 'Tracked changes' in Word files as these are lost in PDF conversion.

I should be grateful if you would also provide a point-by-point response detailing how you have dealt with the points raised by the reviewers in the 'Response to Reviewers' box. Please attend to all of the reviewers' comments. If you do not agree with any of their criticisms or suggestions please explain clearly why this is so.

© 2022. Published by The Company of Biologists under the terms of the Creative Commons Attribution License (https://creativecommons.org/licenses/by/4.0/).

Reviewer 1

Advance summary and potential significance to field

The authors have examined the impact of overexpressing ER-PM tethers in HEK cells on how it changes the nanoscale arrangement of the contact geometry. They then combined this with manipulations of SOCE as well as measures of Ca2+ fluxes from the ER and through the plasma membrane to determine how the same manipulations impacted these parameters. As it stands the data are largely phenomenological and don't offer much in the way of compelling molecular mechanistic insights. I think the work will be useful for the field, as tools like Mapper (S or L), as demonstrated here clearly have functional and and ultrastructural impact on SOCE on ER-PM contacts. As these tools have been used by a number of groups since their introduction. this work serves as a useful benchmark. It is not as easy to interpret the changes in SOCE observed after expressing MAPPER-L. Although the authors favor and exclusion mechanism, the approaches used here are quite indirect and caution is warranted.

Comments for the author

One issue that I think needs attention is a control for the use of Mn2+ as a probe for SOCE via quenching of FURA-2. As the authors use quenching rates as a quantitative measure of ORAI flux, I think it would be important to show that Mn2+ under these conditions is not getting in via some other pathway. Although it is clear that Mn2+ does permeate ORAI and quench Fura, it is unclear in a HEK cell if this might be contaminated by other routes of entry. Whatever control is used needs to be used in for the various conditions examined (such as Mapper-L) as well as controls. What is potentially difficult to understand is why the actual decay rate measured from the slope (fig 7C CA) in the blue (Mapper-S) and black (control) traces look mostly like there is a delay in the start of the decay, but other than that the curves look relatively parallel.

Reviewer 2

Advance summary and potential significance to field

In this study, Henry et al. used electron microscopy and Ca2+ imaging as their primary methods to investigate the impact of the overexpression of various endoplasmic reticulum (ER)-plasma membrane (PM) tethers in the morphological features of ER-PM contacts before and during Store-Operated Ca2+ Entry (SOCE) induced by ER Ca2+ depletion.

They first analyzed untrasfected control cells (HEK293T) and found that ER C2+ depletion causes elongation of the length of ER-PM contacts without changing their gap distance. They then went on to analyze cells that overexpressed various tethers, including E-Syt1, E-Syt2, MAPPER-S, and MAPPER-L.
These tethers did not change the size/length of ER-PM contacts nor their gap distance at steady state. In contrast, upon ER Ca2+ depletion, most of the cells expressing these tethers showed more elongated ER-PM contacts with uniquely fixed gap distance (e.g., 12.8 nm for E-Syt1, 14.5 nm for E-Syt2, 6.4 nm for MAPPER-S, and 8.5 nm for MAPPER-L). Ca2+ influx somewhat reversed the elongation of ER-PM contacts (but not to the steady state level).

Using Ca2+ imaging, they found that overexpression of E-Syt2 and MAPPER-L inhibited SOCE in HEK293T cells (and also in MEF and HeLa cells albeit with slightly different magnitudes depending on an overexpressed tether). Dominant negative calmodulin enhanced SOCE equally in control untreansfected cells and cells overexpressing MAPPER-S or MAPPER-L, and thus the mechanisms of SOCE regulation are maintained even when these tethers are overexpressed. The rate of Mn2+ entry during SOCE (through SOCE channels) was enhanced in control untreansfected cells but not in cells overexpressing MAPPER-S or MAPPER-L, showing the correlation of the hyper elongation of ER-PM contacts induced by these tethers and SOCE inhibition.

Overall, this is an interesting study showing the importance of the proper morphological features of ER-PM contacts for efficient SOCE upon ER Ca2+ depletion. However, there are some major limitations in this study. All the experiments, except experiments with control nontransfected

© 2022. Published by The Company of Biologists under the terms of the Creative Commons Attribution License (https://creativecommons.org/licenses/by/4.0/).

cells, were performed under overexpression of tethers, thus providing limited insights into physiological significance of their findings.
Although the electron micrographs and morphometry are executed very well, additional live cell imaging experiments are needed to provide more mechanistic insights into the effect of the tethers.

Comments for the author

Major Comments:
1. All the experiments were performed under overexpression of tethers. For example, what is the effect of depletion of E-Syts in the morphological features of ER-PM contacts before and during Store-Operated Ca2+ Entry (SOCE)? Do you see enhancement of SOCE upon depletion of E-Syts (in contrast to their overexpression)?
Previous studies performed in Jurkat T cells (PMID: 32879390) showed that depletion of E-Syt1 and E-Syt2 inhibits SOCE, which may go against the model proposed by the authors in the current study. This point/discrepancy needs to be clarified and discussed.
2. The model proposed in Figure 8 is too speculative. It would be necessary to image during SOCE preferably via TIRF, 1) the localization of STIM/ORAI with or without overexpression of the tethers and 2) the localization of the tethers. This will help to better understand the mechanisms and may also support the proposed model.
3. Related to the point #2, it is known that depending on the length of tethers, STIM-ORAI complexes could be in the center or in the periphery of ER-PM contacts regardless of the extent of the elongation of ER-PM contacts (see PMID: 17684017 for example). What will happen to the morphology of ER-PM contacts and SOCE if a tether with a particularly long linker is overexpressed? It may still enhance the elongation of ER-PM contacts, but it may not inhibit SOCE.
4. As mentioned above, some tethers may promote the coupling of STIM-ORAI for maintaining robust SOCE, while other tethers may inhibit it (by possibly pushing them to the periphery). Thus, it is premature to generally conclude "…cER expansion mediated by ER-PM tethering negatively regulate SOCE by confining STIM-ORAI complexes to the periphery of enlarged cER sheets…" as stated in the abstract.
5. FACS enrichment of the transfected cells most likely resulted in selecting cells with low to moderate overexpression. This point should be discussed. In severely overexpressed conditions, the gap distance of ER-PM contacts may be fixed even at non-treated conditions.
6. The statement in the discussion "These data indicate that cER tethering reduces the activity of STIM-ORAI complexes formed upon store depletion and greatly amplifies their Ca2+-dependent inhibition" (page 13) seems to be an overstatement. It is not even clear whether the same amount of STIM is recruited to ER-PM contacts when tethers are overexpressed (please see the point #2).

Minor Comments:
1. Bar graphs should be replaced to dot plots to show the variabilities of the values wherever appropriate.
2. Cite a reference that shows that data in Figure 6C are related to PMCA activities. Isn't this the combination of ER Ca2+ uptake by SERCA and Ca2+ efflux mediated by PMCA?
3. Why is there quenching effect of Fura-2 with Mn2+ even without SOCE induction? Is Mn2+ coming into the cells with ion channels other than ORAI at steady state?
4. Is there an evidence to support that the localization of IP3 receptors is affected by overexpression of tethers? It is stated "cER enlargement negatively regulates Ca2+ release and SOCE, likely by sequestering STIM-ORAI complexes and possibly InsP3 receptors at the periphery of the enlarged cER sheets." It would be good if the authors cite a relevant paper (e.g., PMID: 27591258), which shows the implication of IP3 receptor in this process here in the discussion.

© 2022. Published by The Company of Biologists under the terms of the Creative Commons Attribution License (https://creativecommons.org/licenses/by/4.0/).

First revision

Author response to reviewers' comments

Rebuttal letter (reviewers' comments are in italics, our responses in plain letters)

Dear Editors,

We thank the reviewers for the critical evaluation of our work. The following is a point-by-point response and any reference to pages and line number corresponds to the word file containing all the tracked changes.

Reviewer 1 Advance Summary and Potential Significance to Field:

The authors have examined the impact of overexpressing ER-PM tethers in HEK cells on how it changes the nanoscale arrangement of the contact geometry. They then combined this with manipulations of SOCE as well as measures of Ca2+ fluxes from the ER and through the plasma membrane to determine how the same manipulations impacted these parameters. As it stands the data are largely phenomenological and don't offer much in the way of compelling molecular mechanistic insights. I think the work will be useful for the field, as tools like Mapper (S or L), as demonstrated here, clearly have functional and and ultrastructural impact on SOCE on ER-PM contacts. As these tools have been used by a number of groups since their introduction. this work serves as a useful benchmark. It is not as easy to interpret the changes in SOCE observed after expressing MAPPER-L. Although the authors favor and exclusion mechanism, the approaches used here are quite indirect and caution is warranted.

We thank the reviewer for the insightful comments and suggestions. We agree that caution is warranted in interpreting the SOCE changes induced by MAPPER-L and have performed additional live cell imaging experiments to confirm that an exclusion mechanism likely accounts for the functional defects reported.

Reviewer 1 Comments for the Author:

One issue that I think needs attention is a control for the use of Mn2+ as a probe for SOCE via quenching of FURA-2. As the authors use quenching rates as a quantitative measure of ORAI flux, I think it would be important to show that Mn2+ under these conditions is not getting in via some other pathway. Although it is clear that Mn2+ does permeate ORAI and quench Fura, it is unclear in a HEK cell if this might be contaminated by other routes of entry. Whatever control is used needs to be used in for the various conditions examined (such as Mapper-L) as well as controls. What is potentially difficult to understand is why the actual decay rate measured from the slope (fig 7C CA) in the blue (Mapper-S) and black (control) traces look mostly like there is a delay in the start of the decay, but other than that the curves look relatively parallel.

We have addressed this point experimentally. The new Fig. S7B shows that Mn^{2+} quenching is abrogated in HEK-293 cells lacking all three Orai isoforms (TKO cells). mCh-Orai1 expression in TKO cells restored Mn^{2+} fluxes, indicating that the Mn^{2+} quench assay primarily reports SOCE mediated by the Orai1 channel. Importantly, MAPPER-L expression reduced Mn^{2+} influx to the same extent in wild-type cells and in TKO cells expressing mCh-Orai1 (25% vs. 21% inhibition, respectively, Fig. 7B). These data indicate that Mn^{2+} enters HEK-293 cells predominantly via Orai1 channels, whose activity is decreased by enforced ER tethering. We thank the reviewer for suggesting this important control experiment.

The apparent delay in the blue curve in Fig. 7C CA reflects the slow onset of Mn^{2+} quenching in this specific condition. We did not consider this lag time in our analysis and only measured the maximal quench rates as fura-2 fluorescence decreased linearly during the first minute following Mn^{2+} addition. The maximal quench rates were significantly reduced in the MAPPER-S (blue) condition, but we agree that the difference in slope was not apparent in the 3 min recordings shown in Fig.

© 2022. Published by The Company of Biologists under the terms of the Creative Commons Attribution License (https://creativecommons.org/licenses/by/4.0/).

7C. We have rescaled these recordings to show only the first minute following Mn^{2+} addition. We hope that this presentation better illustrates the inhibitory effect of MAPPER expression on Mn^{2+} entry.

Reviewer 2 Advance Summary and Potential Significance to Field:
In this study, Henry et al. used electron microscopy and Ca2+ imaging as their primary methods to investigate the impact of the overexpression of various endoplasmic reticulum (ER)-plasma membrane (PM) tethers in the morphological features of ER-PM contacts before and during Store-Operated Ca2+ Entry (SOCE) induced by ER Ca2+ depletion.

They first analyzed untrasfected control cells (HEK293T) and found that ER C2+ depletion causes elongation of the length of ER-PM contacts without changing their gap distance. They then went on to analyze cells that overexpressed various tethers, including E-Syt1, E-Syt2, MAPPER-S, and MAPPER-L. These tethers did not change the size/length of ER-PM contacts nor their gap distance at steady state. In contrast, upon ER Ca2+ depletion, most of the cells expressing these tethers showed more elongated ER- PM contacts with uniquely fixed gap distance (e.g., 12.8 nm for E-Syt1, 14.5 nm for E-Syt2, 6.4 nm for MAPPER-S, and 8.5 nm for MAPPER-L). Ca2+ influx somewhat reversed the elongation of ER-PM contacts (but not to the steady state level).

Using Ca2+ imaging, they found that overexpression of E-Syt2 and MAPPER-L inhibited SOCE in HEK293T cells (and also in MEF and HeLa cells albeit with slightly different magnitudes depending on an overexpressed tether). Dominant negative calmodulin enhanced SOCE equally in control untreansfected cells and cells overexpressing MAPPER-S or MAPPER-L, and thus the mechanisms of SOCE regulation are maintained even when these tethers are overexpressed. The rate of Mn2+ entry during SOCE (through SOCE channels) was enhanced in control untreansfected cells but not in cells overexpressing MAPPER-S or MAPPER-L, showing the correlation of the hyper elongation of ER-PM contacts induced by these tethers and SOCE inhibition.

Overall, this is an interesting study showing the importance of the proper morphological features of ER- PM contacts for efficient SOCE upon ER Ca2+ depletion. However, there are some major limitations in this study. All the experiments, except experiments with control nontransfected cells, were performed under overexpression of tethers, thus providing limited insights into physiological significance of their findings. Although the electron micrographs and morphometry are executed very well, additional live cell imaging experiments are needed to provide more mechanistic insights into the effect of the tethers.

We thank the reviewer for the careful consideration or our work and the insightful suggestions. We have performed the suggested live cell imaging experiments. The results show that the expressed tethers are juxtaposed to STIM-ORAI complexes, consistent with the exclusion mechanism that we propose.

Reviewer 2 Comments for the Author:
Major Comments:

1. All the experiments were performed under overexpression of tethers. For example, what is the effect of depletion of E-Syts in the morphological features of ER-PM contacts before and during Store-Operated Ca2+ Entry (SOCE)? Do you see enhancement of SOCE upon depletion of E-Syts (in contrast to their overexpression)?

We have addressed this point experimentally. The Fig. R1A below shows that E-Syt1 mRNA levels are low in HEK-293 cells and not further decreased by si-RNAs silencing, while HEK-293 E-Syt2 mRNA levels averaged 72% of Jurkat T cells and decreased by 50% with a si-RNA targeting E-Syt2. These two si-RNAs had no impact on SOCE, probably due to the low endogenous expression levels of these proteins in HEK- 293 cells (Fig. R1B). These data are consistent with an earlier report that E-Syt1/2 depletion does not impact SOCE in HeLa cells or the length of cortical ER structures in Jurkat cells {Woo, 2020 #92} and suggest that endogenous levels of E-Syts do not contribute significantly to the elongation of the cortical ER occurring during store depletion in HEK-293 cells.

© 2022. Published by The Company of Biologists under the terms of the Creative Commons Attribution License (https://creativecommons.org/licenses/by/4.0/).

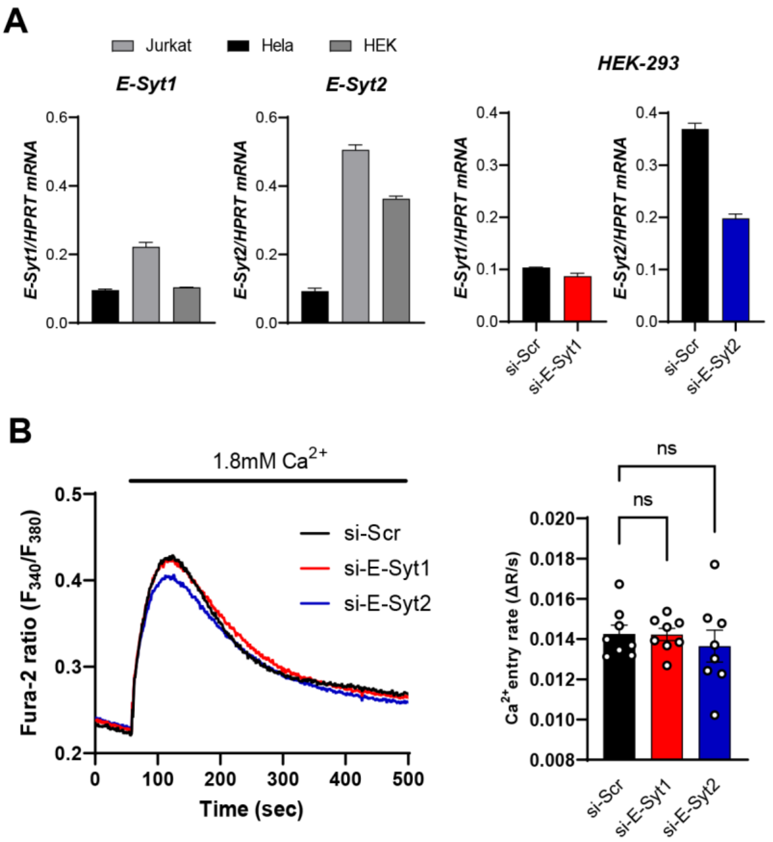

Figure R1: **E-Syt1 &2 silencing does not alter SOCE**. A. mRNA levels of E-Syt1 and E-Syt2 in Hela, Jurkat T cells, and HEK-293 cells exposed or not to the indicated si-RNAs, relative to the housekeeping gene hypoxanthine guanine phosphoribosyl transferase (HPRT). B. Fura-2 recordings (left) and Ca^{2+} entry rates (right) evoked by Tg/Ca^{2+} readmission in HEK-293 cells expressing the indicated si-RNA.

Previous studies performed in Jurkat T cells (PMID: 32879390) showed that depletion of E-Syt1 and E- Syt2 inhibits SOCE, which may go against the model proposed by the authors in the current study. This point/discrepancy needs to be clarified and discussed.

While depletion of both E-Syt1 and E-Syt2 inhibits SOCE in Jurkat T cells, this effect was not observed in HeLa cells {Woo, 2020 #92} and our si-RNAs targeting these two isoforms did not inhibit SOCE in HEK- 293 cells (Fig. R1). This might reflect the expression of distinct isoforms in immune and non-immune cells, with the activating isoform E-Syt2b possibly accounting for the inhibitory effects of E-Syt1/2 silencing in Jurkat T cells. These points are discussed in the revised MS (p. 12).

2. *The model proposed in Figure 8 is too speculative. It would be necessary to image during SOCE, preferably via TIRF, 1) the localization of STIM/ORAI with or without overexpression of the tethers and 2) overexpression of the tethers. This will help to better understand the mechanisms and may also support the proposed model.*

We have performed the suggested experiments. The new Fig. 8A shows TIRF images of cells co-expressing CFP-STIM1 and mCh-Orai1 with or without GFP-MAPPER-L. GFP-MAPPER-L formed clusters juxtaposed to STIM-ORAI sites as can be appreciated from line-scan drawn on the inset of the merged image. The localization as well as the morphometric parameters of STIM1/ORAI1 clusters (co- localization coefficient, fraction of the PM decorated, and area of individual clusters) were not altered by the expression of MAPPER-L (Fig. S8A & B). These data indicate that the tethers accumulate in cortical ER structures adjacent to STIM/ORAI interaction sites as proposed in our model in Figure 8. We thank the reviewer for suggesting this experiment.

© 2022. Published by The Company of Biologists under the terms of the Creative Commons Attribution License (https://creativecommons.org/licenses/by/4.0/).

3. *Related to the point #2, it is known that depending on the length of tethers, STIM-ORAI complexes could be in the center or in the periphery of ER-PM contacts regardless of the extent of the elongation of ER-PM contacts (see PMID: 17684017 for example). What will happen to the morphology of ER-PM contacts and SOCE if a tether with a particularly long linker is overexpressed? It may still enhance the elongation of ER-PM contacts, but it may not inhibit SOCE.*

We have performed the suggested experiments, using the SNARE protein Sec22b and its extended version Sec22b-P33 to anchor the ER at 20 and 30 nm from the PM, respectively {Petkovic, 2014 #93}. The new Fig. S8C shows that these two long tethers co-localize with STIM/ORAI clusters in the TIRF plane, in sharp contrast to MAPPER-L. Fig. S8D further shows that their overexpression does not impact SOCE. We postulate that the distinct distribution of the two tethers reflects the exclusion of STIM-bound ORAI1 from cortical ER structures with narrow gaps. We thank the reviewer for suggesting the experiment which strengthen our model.

4. *As mentioned above, some tethers may promote the coupling of STIM-ORAI for maintaining robust SOCE, while other tethers may inhibit it (by possibly pushing them to the periphery). Thus, it is premature to generally conclude "…cER expansion mediated by ER-PM tethering negatively regulate SOCE by confining STIM-ORAI complexes to the periphery of enlarged cER sheets…" as stated in the abstract.*

We agree that this conclusion was premature, particularly given the results from the additional experiments detailed above showing that enforced tethering only inhibits SOCE when short tethers are used. We have now rephrased our abstract to specify that ER tethering **at a close distance** negatively regulates SOCE (line 13).

5. *FACS enrichment of the transfected cells most likely resulted in selecting cells with low to moderate overexpression. This point should be discussed. In severely overexpressed conditions, the gap distance of ER-PM contacts may be fixed even at non-treated conditions.*

Good point. We now specify that cells with low to moderate overexpression were selected by the FACS enrichment procedure to avoid the formation of artificial cortical ER structures in the absence of store depletion (p. 7). Expressing tether proteins at high levels does indeed generate cortical ER structures in the absence of store depletion, and we observed that MAPPER-L generates long cortical ER structures in STIM1/2-deficient (DKO) cells (Fig. R2 below). This indicates that the MAPPERs can tether and elongate ER-PM junctions independently of the store-operated machinery.

© 2022. Published by The Company of Biologists under the terms of the Creative Commons Attribution License (https://creativecommons.org/licenses/by/4.0/).

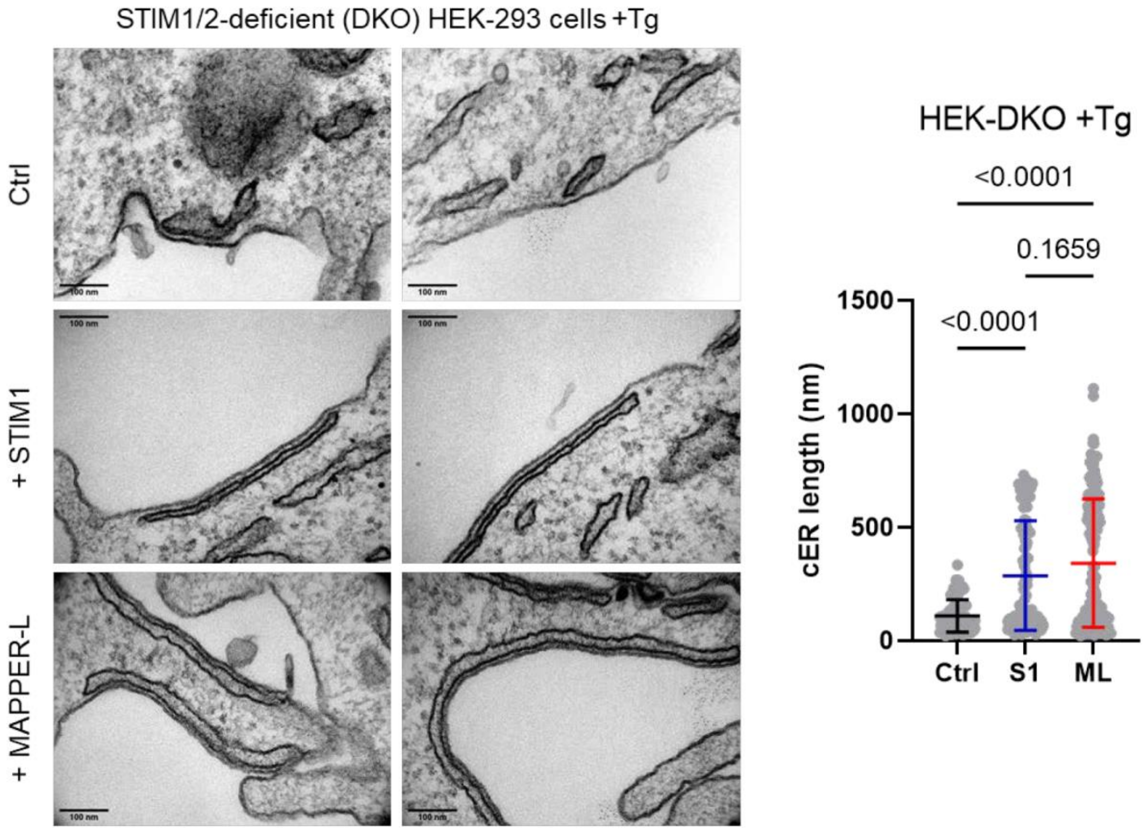

Figure R2. ***Effect of STIM1 and MAPPER-L expression in STIM1/2-deficient cells***. Left: Representative electron micrographs of cER sheets in STIM1/2-deficient (double KO, DKO) HEK-293 cells expressing the indicated constructs and treated with Tg for 10 minutes in Ca^{2+}-containing medium. Right: Dot plots of cER length in each condition. STIM1 and MAPPER-L expression both induced the apparition of elongated cER sheets in cells lacking endogenous STIM proteins.

6. The statement in the discussion "These data indicate that cER tethering reduces the activity of STIM- ORAI complexes formed upon store depletion and greatly amplifies their Ca2+-dependent inhibition" (page 13) seems to be an overstatement. It is not even clear whether the same amount of STIM is recruited to ER-PM contacts when tethers are overexpressed (please see the point #2).

We agree and have rephrased this statement to omit the mention of a Ca^{2+}-dependent inhibition of signaling molecules. The new sentence (p. 14) reads: "These data indicate that enforced cER elongation limits SOCE, suggesting that part of the elongated cER structures do not favor STIM/ORAI interactions. Accordingly, STIM/ORAI clusters were juxtaposed with MAPPER-L in the TIRF plane (Fig. 8B)."

Minor Comments:

1. Bar graphs should be replaced to dot plots to show the variabilities of the values wherever appropriate. We have replaced 16 bar graphs panels by dot blots and retained four panels with large numbers of samples and conditions that are better illustrated as bar graphs.

2. Cite a reference that shows that data in Figure 6C are related to PMCA activities. Isn't this the combination of ER Ca2+ uptake by SERCA and Ca2+ efflux mediated by PMCA?

This experiment was performed in the presence of thapsigargin, thus SERCA are inhibited, and the efflux rates reflect the activity of plasma membrane extrusion mechanisms, essentially PMCA with a possible contribution of NCX. We now cite an earlier reference for this protocol {Frieden, 2005 #88}.

© 2022. Published by The Company of Biologists under the terms of the Creative Commons Attribution License (https://creativecommons.org/licenses/by/4.0/).

3. Why is there quenching effect of Fura-2 with Mn2+ even without SOCE induction? Is Mn2+ coming into the cells with ion channels other than ORAI at steady state?

We now show that fura-2 quenching by Mn^{2+} is abrogated in Orai1/2/3-deficient TKO cells and restored by Orai1 re-expression, confirming that the fluxes are mediated by Orai1 (new Fig. S7B and our response to reviewer 1). The basal quench rates of non-treated WT HEK-293 cells likely reflect the constitutive activity of endogenous Orai channels, which increased by about 2-fold upon store depletion.

4. Is there an evidence to support that the localization of IP3 receptors is affected by overexpression of tethers? It is stated "cER enlargement negatively regulates Ca2+ release and SOCE, likely by sequestering STIM-ORAI complexes and possibly InsP3 receptors at the periphery of the enlarged cER sheets." It would be good if the authors cite a relevant paper (e.g., PMID: 27591258), which shows the implication of IP3 receptor in this process here in the discussion.

We now cite this paper and recent publications reporting that IP3R immobilized at ER-PM junctions form a functional unit for SOCE {Thillaiappan, 2017 #90;Thillaiappan, 2021 #91}.

<u>Second decision letter</u>

MS ID#: JOCES/2021/259313

MS TITLE: Enforced tethering elongates the cortical endoplasmic reticulum and limits store-operated calcium entry

AUTHORS: Christopher Henry, Amado Carreras Sureda, and Nicolas Demaurex
ARTICLE TYPE: Research Article

I am happy to tell you that your manuscript has been accepted for publication in Journal of Cell Science, pending standard ethics checks.

© 2022. Published by The Company of Biologists under the terms of the Creative Commons Attribution License (https://creativecommons.org/licenses/by/4.0/).
